# The impact of health insurance on hypertension care: a household fixed effects study in India

**DOI:** 10.1186/s12889-024-19759-1

**Published:** 2024-08-22

**Authors:** Zixuan Feng, Qiushi Chen, Lirui Jiao, Xuedi Ma, Rifat Atun, Pascal Geldsetzer, Till Bärnighausen, Simiao Chen

**Affiliations:** 1 The Harold and Inge Marcus Department of Industrial and Manufacturing Engineering, The Pennsylvania State University, University Park, PA USA; 2 Department of Health Policy and Management, Gillings School of Global Public Health, University of North Carolina at Chapel Hill, Chapel Hill, NC USA; 3 Chinese Academy of Medical Sciences and Peking Union Medical College, Beijing, China; 4 Harvard T.H. Chan School of Public Health, Harvard University, Boston, MA USA; 5 Division of Primary Care and Population Health, Stanford University, Stanford, CA USA; 6 Chan Zuckerberg Biohub, San Francisco, CA USA; 7 Faculty of Medicine and University Hospital, Heidelberg Institute of Global Health, Heidelberg University, Heidelberg, Germany

**Keywords:** Hypertension, Health insurance, Care cascade, Health policy, Unmet needs

## Abstract

**Introduction:**

Hypertension is highly prevalent in India, but the proportion of patients achieving blood pressure control remains low. Efforts have been made to expand health insurance coverage nationwide with the aim of improving overall healthcare access. It is critical to understand the role of health insurance coverage in improving hypertension care.

**Methods:**

We used secondary data from the nationally representative sample of adults aged 15–49 years from the 2015–2016 National Family Health Survey (NFHS) in India. We defined the hypertension care cascade as four successive steps of (1) screened, (2) diagnosed, (3) treated, and (4) controlled, and operationalized these variables using blood pressure measurements and self-reports. We employed household fixed effect models that conceptually matched people with and without insurance within the household, to estimate the impact of insurance coverage on the likelihood of reaching each care cascade step, while controlling for a wide range of additional individual-level variables.

**Results:**

In all 130,151 included individuals with hypertension, 20.4% reported having health insurance. For the insured hypertensive population, 79.8% (95% Confidence Interval: 79.3%-80.3%) were *screened*, 49.6% (49.0%-50.2%) *diagnosed*, 14.3% (13.9%-14.7%) *treated*, and 7.9% (7.6%-8.2%) *controlled*, marginally higher than the percentages for the uninsured 79.8% (79.5%-80.0%), 48.2% (47.9%-48.6%), 13.3% (13.1%-13.5%), and 7.5% (7.4%-7.7%) for each cascade step, respectively. From the household fixed effects model, health insurance did not show significant impact on the hypertension care cascade, with the estimated relative risks of health insurance 0.97 (0.93–1.02), 0.97 (0.91–1.03), 0.95 (0.77–1.30), and 0.97 (0.65–1.10) for each cascade step, respectively. We further performed stratified analyses by sociodemographic and behavioral risk factors and a sensitivity analysis with district fixed effects, all of which yielded results that confirmed the robustness of our main findings.

**Conclusions:**

Health insurance did not show significant impact on improving hypertension care cascade among young and middle-aged adults with hypertension in India. Innovative strategies for overcoming practical barriers to healthcare services in addition to improving financial access are needed to address the large unmet need for hypertension care.

**Supplementary Information:**

The online version contains supplementary material available at 10.1186/s12889-024-19759-1.

## Introduction

Cardiovascular disease (CVD) accounted for one-third of all deaths worldwide in 2015 [1] and is the leading cause of death in India [2]. The CVD age-standardized mortality rate in India was estimated to be 15.7% higher than the global average in 2010 [2]. Hypertension, which is a major risk factor for CVD [3], is common in India, with an estimated prevalence of 20.6% in males and 20.9% in females in 2000; these figures were projected to increase to 22.9% and 23.6%, respectively, by 2025 [4]. India also faces one of the highest economic burdens due to CVD among all low- and middle-income countries [5]. Despite the growing hypertensive population and its substantial implications for population health and economic well-being, the management of hypertension in India remains suboptimal. A nationally representative study in 2015–2016 on hypertension care revealed that only 7.9% of the hypertensive population had their blood pressure *controlled* with medication [6], which is well below the global average of 10.3% [7].

As India undergoes demographic and epidemiological transitions, the country needs effective strategies to address its health burdens, including the management of chronic diseases such as hypertension [8]. One common approach to improving healthcare access is through health insurance [9, 10]. Previous literature has shown positive impacts of health insurance on various hypertension management outcomes, such as improving blood pressure monitoring and adherence to antihypertensive medications [11]. Studies have also found that insurance can encourage patients to maintain regular care [12], and thus to improve hypertension treatment and control [13]. In addition, achieving hypertension control is more likely for newly insured patients than the continuously uninsured, continuously insured, and discontinuously insured patients, and patients living in the most deprived neighborhoods may experience the greatest benefit from health insurance [14].

However, most prior studies have only examined the effects of health insurance on specific hypertension care outcomes [11, 15], rather than taking a comprehensive view of the entire continuum of care, which is typically referred to as a *care cascade*. While the care cascade model was first utilized to address gaps in the HIV care continuum from diagnosis to viral suppression [16, 17], the concept has been generalized and is now commonly used to systematically study healthcare delivery for other chronic diseases, such as tuberculosis [18, 19], diabetes [20], and chronic hepatitis C infection [21, 22]. Understanding the care cascade for a given disease is critical for quantifying the quality of care delivery, assessing unmet needs, and identifying care gaps to inform suitable programmatic and policy responses [23, 24]. Hypertension care cascade outcomes have previously been evaluated in various settings [6, 7, 16, 25], but data showing the potential impact of health insurance on the hypertension care cascade remain limited.

To bridge this knowledge gap, we examined the impact of health insurance coverage on hypertension care cascade outcomes in India, leveraging nationally representative health survey data. Unlike most previous studies examining the association of health insurance and hypertension care outcomes with simple analytical models [26–28], we employed household fixed effects models, a commonly used study design in public health research, to produce stronger evidence for policy formulation [29–32]. In this study, our objective was to evaluate the impact of health insurance on improving hypertension care, which is one of the largest unmet healthcare needs in India and globally, thereby informing the development of effective strategies for improving healthcare access and health outcomes among patients with hypertension. In addition, as the Indian government promotes health insurance to improve healthcare access, in particular for the country’s poorest populations, we also analyzed the impact by wealth groups to provide further insights in the impact of health insurance for India’s most financially vulnerable populations.

## Methods

### Data source

We used the secondary data obtained from the 2015–2016 National Family Health Survey (NFHS-4) with nationally representative samples in India [33, 34] to extract individual-level information on hypertension management, health insurance coverage, demographic and health-related variables. We used the NFHS-4 data that were collected prior to the wider rollout of public health insurance schemes for household coverage (e.g., India’s National Health Policy 2017 and The Ayushman Bharat Program [35–37]), which allowed us to employ the household fixed effects design (see details below) to draw stronger evidence for the impact of health insurance on hypertension care cascade. This household-based survey was conducted by the Ministry of Health and Family Welfare in India and collected demographic, health, and nutrition data for the sampled population, composed of 112,122 males aged 15 to 54 years and 699,686 females aged 15 to 49 years from 601,509 households in all states and union territories in India [33]. NFHS-4 followed a stratified two-stage sampling approach for systematically selecting primary sampling units (PSUs), sampling clusters within each PSU, and sampling households from each cluster [33]. The survey collected household-level information (e.g., urban vs. rural residency and household wealth), individual-level information (e.g., demographics, education level, and marital status), and sex-specific information (e.g., fertility, childcare, and domestic violence). Biomarkers were also collected, including measurements of height, weight, hemoglobin level, blood pressure, and blood glucose level [33]. Our analysis focused on males and females aged 15–49 years old and excluded samples with missing variable values, inconsistent responses, or abnormal blood pressure readings.

### Hypertension care cascade outcome measures

NFHS-4 encompassed a comprehensive interview regarding health status for all survey participants. We identified the hypertensive cohort of interest to this study based on either of the following two criteria: (1) having a raised blood pressure reading or (2) having a previous diagnosis of hypertension. For the first criterion, we determined blood pressure was raised if systolic blood pressure was above 140 mm Hg or diastolic blood pressure was above 90 mm Hg, according to the Indian Hypertension Guidelines published in 2013 [38]. In NFHS-4, interviewers were instructed to measure each individual’s blood pressure (using an Omron Blood Pressure Monitor) three times over a single visit, with at least a five-minute interval between readings. Some individuals lacked complete data, with 3.4% of participants missing at least two measurements. If multiple values for an individual were available, the average of these readings was used in our analysis. Individuals for whom all blood pressure readings were missing or who had abnormal readings (i.e., < 20 mm Hg or > 300 mm Hg) were excluded from the analysis. In addition, pregnant females were excluded from our study cohort since pregnancy may lead to abnormal blood pressure readings [39].

For the second criterion, having a previous diagnosis of hypertension was defined based on the answer of “yes” to both of the following questions: (1) “Were you told on 2 or more different occasions by a doctor or other health professionals that you had hypertension or high blood pressure?” and (2) “To lower your blood pressure, are you now taking a prescribed medicine?” Individuals with inconsistent answers (i.e., “no” to question (1) but “yes” to question (2)) were excluded from the study cohort.

For the cohort with hypertension as identified above, we defined the hypertension care cascade as encompassing the successive steps of *screened, diagnosed, treated, and controlled*. Individuals were considered to have been *screened* if they answered “yes” to the survey question “Before this survey, has your blood pressure ever been checked?” Individuals were considered to have been *diagnosed* if they answered “yes” to either of the following two questions: (1) “Were you told on two or more different occasions by a doctor or other health professional that you had hypertension or high blood pressure?” or (2) “To lower your blood pressure, are you now taking a prescribed medicine?” Individuals who answered “yes” to the second question were considered as *treated.* Lastly, individuals were considered to have had their hypertension *controlled* if their average blood pressure values were within the normal ranges after being *treated*.

### Health insurance coverage and covariates

We determined that sampled individuals had (self-reported) health insurance coverage if they responded “yes” to the question “Are you covered by any health insurance?” We further validated each individual’s health insurance coverage status by examining their response to the question “What type of health insurance are you covered by?” which had nine different types of insurance as possible responses. Individuals with inconsistent responses (i.e., those who answered “no” to the first question but still selected one of the nine insurance types for the second question) were to be excluded from the analysis; however, no inconsistent responses were observed.

Individual-level demographic variables such as age, sex, education level, and marital status; individual-level health variables such as body mass index (BMI) and tobacco consumption; and household-level variables such as urban vs. rural residence and household wealth were included in our statistical analysis. Age, initially recorded as a continuous variable in NFHS-4, was regrouped into five-year age groups ranging from 15 to 49. Education level was categorized as “no education,” “primary school unfinished,” “primary school finished,” “secondary school unfinished,” “secondary school finished,” or “above secondary school.” The BMI values were calculated using height and weight variables and were categorized as < 18.5 kg/m^2^ (thin), 18.5–24.9 kg/m^2^ (normal), 25.0–29.9 kg/m^2^ (overweight), and > 30 kg/m^2^ (obese), according to the NFHS-4 User Manual [33]. Tobacco consumption included two separate binary variables, “current smoker” and “uses smokeless tobacco,” defined by aggregating multiple smoking and lifestyle-related survey questions [6]. Urban vs. rural residence status (for households) was directly available from the survey data. To determine household wealth, we obtained the wealth index factor score for each household, which was recorded in NFSH-4 as a five-digit value representing the number of different kinds of consumer goods owned by the household and housing characteristics (International Institute for Population, 2017). The scores were then used to categorize households into the lower, middle, and upper tertiles (based on the 33% and 67% percentiles).

### Statistical analysis

We estimated the proportion of individuals reaching each care cascade step (*screened*, *diagnosed*, *treated*, and *controlled*) among the hypertensive population stratified by self-reported insurance coverage status. To evaluate the impact of health insurance coverage on the likelihood of reaching each care cascade step, we applied the household fixed effects model, a study design that has previously been used to yield strong evidence of policy impact and establish potential causal relationships [30]. Specifically, we considered the household fixed effects to control for unobserved factors that were common for members within a household and that may have potentially influenced healthcare utilization patterns and health outcomes, such as living and dietary habits and access to transportation services connecting the residence to healthcare facilities [19]. In the regression model for each step of the hypertension care cascade, the dependent variable was defined as whether a given cascade step (i.e., *screened, diagnosed, treated,* or *controlled*) was reached, and all individuals who had reached the previous care cascade step were included in the sample. For example, for the regression model with the *diagnosed* cascade step as the dependent variable, the sample included all individuals who had reached the *screened* step of the care cascade. We chose modified Poisson regression for dichotomous dependent variables [40, 41] because the estimated relative risk (RR) provided a consistent estimate of the average effect and was deemed easier to interpret than an odds ratio estimated from logistic regression [41]. The modified Poisson regression model specification is as follows:

$$\text{log}\left(E\left[{Y}_{ih}\right]\right)=\beta \cdot {\text{Insured}}_{ih}+{\varvec{\gamma}}{{\prime}{\varvec{X}}}_{ih}+{\mu }_{h},$$

where $E({Y}_{ih})$ represents the expected outcome (or the probability) of whether individual $i$ in household $h$ reaches a specific cascade step (with ${Y}_{ih}$ following a Poisson distribution); ${\text{Insured}}_{ih}$ represents individual $i$’s insurance coverage status (binary); ${{\varvec{X}}}_{ih}$ represents a collection of the individual’s covariates including sex, age group, education level, marital status, BMI, and tobacco consumption; and ${\mu }_{h}$ represents household fixed effects. The primary outcome of interest is the estimated value of coefficient $\beta$, representing the impact of health insurance coverage on the probability of reaching each step of the hypertension care cascade among individuals with hypertension. The standard errors in our model were adjusted at the primary sampling unit level, as it was the largest sampling unit in the original NFHS-4 survey design [6, 33]. Our regression model accounted for the sample weights from NFHS-4. Considering that individuals’ weights in NFHS-4 were computed separately for males and females with unbalanced proportions, we followed the approach used in the previous study [6] to rescale the males’ individual weights to make the combined sample representative of the general population of India. Specifically, we used the females’ individual weights as the reference and then scaled up the males’ individual weights based on the proportion of males in each single-year age group in the general Indian population per Census Data [42].

To further explore potential differences in the impact of health insurance coverage by major demographic and behavioral subgroups, we performed stratified analyses by sex, age group (≤ 30 vs. > 30 years old), education (secondary school finished and above vs. others), BMI (≥ 25 vs. < 25 kg/m^2^), and smoking behavior (smoker vs. nonsmoker). We also stratified our household fixed effect analysis by urban vs. rural residence and household wealth categories to explore differences in impact size across each of these categories.

To address the limitations of household fixed effects models and assess the robustness of our results, we also analyzed district-level fixed effect models that could utilize the full cohort and directly include urban vs. rural residence status and household wealth as covariates. All data and statistical analyses were performed in R (version 4.1.1), and the fixed effects models were implemented using the “fixest” package version 0.10.1 in R.

## Results

### Cohort baseline characteristics

A summary of the study cohort characteristics is provided in Table 1. After excluding individuals not in the age range of 15–49 years (1.1% of the full NFHS-4 samples of 811,808 individuals), pregnant females (4.0%), and individuals with incomplete or inconsistent responses (2.7%), we obtained a (unweighted) cohort of 748,396 persons including 99,761 (13.3%) males and 648,635 (86.7%) females from 471,496 households in India for our analysis (Figure S1). Among these individuals, 137,065 (18.3%) were covered by health insurance.

**Table 1:** Baseline characteristics of sample from the 2015–2016 National Family Health Survey in India

Number of individuals, N	Overall	Insured	Uninsured	*P*-value^*^
	**748,396**	**137,065**	**611,331**	
**Age (years)**				< 0.01
Mean (SD)	30.1 (9.87)	31.6 (10.0)	29.7 (9.80)	
Median [Min, Max]	29.0 [15.0, 49.0]	32.0 [15.0, 49.0]	29.0 [15.0, 49.0]	
**Age group**				< 0.01
15–19 years	135,816 (18.1%)	21,016 (15.3%)	114,800 (18.8%)	
20–24 years	122,179 (16.3%)	18,410 (13.4%)	103,769 (17.0%)	
25–29 years	117,908 (15.8%)	19,694 (14.4%)	98,214 (16.1%)	
30–34 years	105,015 (14.0%)	19,719 (14.4%)	85,296 (14.0%)	
35–39 years	100,303 (13.4%)	208,20 (15.2%)	79,483 (13.0%)	
40–44 years	858,69 (11.5%)	18,729 (13.7%)	67,140 (11.0%)	
45–49 years	81,306 (10.9%)	18,677 (13.6%)	62,629 (10.2%)	
**Sex**				< 0.01
Male	99,761 (13.3%)	19,812 (14.5%)	79,949 (13.1%)	
Female	648,635 (86.7%)	117,253 (85.5%)	531,382 (86.9%)	
**Residence**				< 0.01
Urban	220,657 (29.5%)	37,533 (27.4%)	183,124 (30.0%)	
Rural	527,739 (70.5%)	99,532 (72.6%)	428,207 (70.0%)	
**Household wealth**				< 0.01
Lower	248,349 (33.2%)	43,896 (32.0%)	204,453 (33.4%)	
Middle	250,641 (33.5%)	48,717 (35.5%)	201,924 (33.0%)	
Upper	249,406 (33.3%)	44,452 (32.4%)	204,954 (33.5%)	
**Diastolic blood pressure (mmHg)**				< 0.01
Mean (SD)	78.0 (12.1)	78.3 (12.1)	77.9 (12.1)	
Median [Min, Max]	77.0 [26.0, 300]	77.3 [33.3, 300]	77.0 [26.0, 300]	
**Systolic blood pressure (mmHg)**				< 0.01
Mean (SD)	116.4 (14.1)	116.2 (14.6)	116.4 (14.0)	
Median [Min, Max]	115.0 [34.3, 300]	115.0 [47.7, 292]	115.0 [34.3, 300]	
**Education Level**				< 0.01
No education	195,803 (26.2%)	34,660 (25.3%)	161,143 (26.4%)	
Primary school unfinished	44,323 (5.9%)	9,966 (7.3%)	34,357 (5.6%)	
Primary school finished	49,997 (6.7%)	9,235 (6.7%)	407,62 (6.7%)	
Secondary school unfinished	301,703 (40.3%)	55,331 (40.4%)	246,372 (40.3%)	
Secondary school finished	67,798 (9.1%)	11,875 (8.7%)	55,923 (9.1%)	
Secondary school above	88,772 (11.9%)	15,998 (11.7%)	72,774 (11.9%)	
**Marital Status**				< 0.01
Unmarried	232,943 (31.1%)	40,183 (29.3%)	192,760 (31.5%)	
Married	515,453 (68.9%)	96,882 (70.7%)	418,571 (68.5%)	
**BMI**				< 0.01
< 18.5 kg/m^2^ (Thin)	446,435 (59.7%)	81,602 (59.5%)	364,833 (59.7%)	
18.5–24.9 kg/m^2^ (Normal)	165,166 (22.1%)	28,416 (20.7%)	136,750 (22.4%)	
25.0–29.9 kg/m^2^ (Overweight)	106,171 (14.2%)	21,032 (15.3%)	85,139 (13.9%)	
> 30.0 kg/m^2^ (Obese)	30,624 (4.1%)	6,015 (4.4%)	24,609 (4.0%)	
**Tobacco Consumption**				
Current smoker	117,350 (15.7%)	26,297 (19.2%)	91,053 (14.9%)	< 0.01
Uses smokeless tobacco	91,023 (12.2%)	19,108 (13.9%)	71,915 (11.8%)	< 0.01
**Hypertension care**				
Has hypertension	130,151 (17.4%)	26,568 (19.4%)	103,583 (16.9%)	< 0.01
Screened	443,601 (59.3%)	83,568 (61.0%)	360,033 (58.9%)	< 0.01
Diagnosed	63,146 (8.4%)	13,175 (9.6%)	49,971 (8.2%)	< 0.01
Treated	17,578 (2.3%)	3,793 (2.8%)	13,785 (2.3%)	< 0.01
Controlled	9,888 (1.3%)	2,106 (1.5%)	7,782 (1.3%)	< 0.01

Summary statistics are not weighted

*SD* Standard deviation, *Min *Minimum, *Max* Maximum, *BMI* Body mass index

^*^P-values were for the comparison of characteristics between insured and uninsured groups

### Impacts of insurance coverage on the hypertension care cascade

**Fig. 1:**
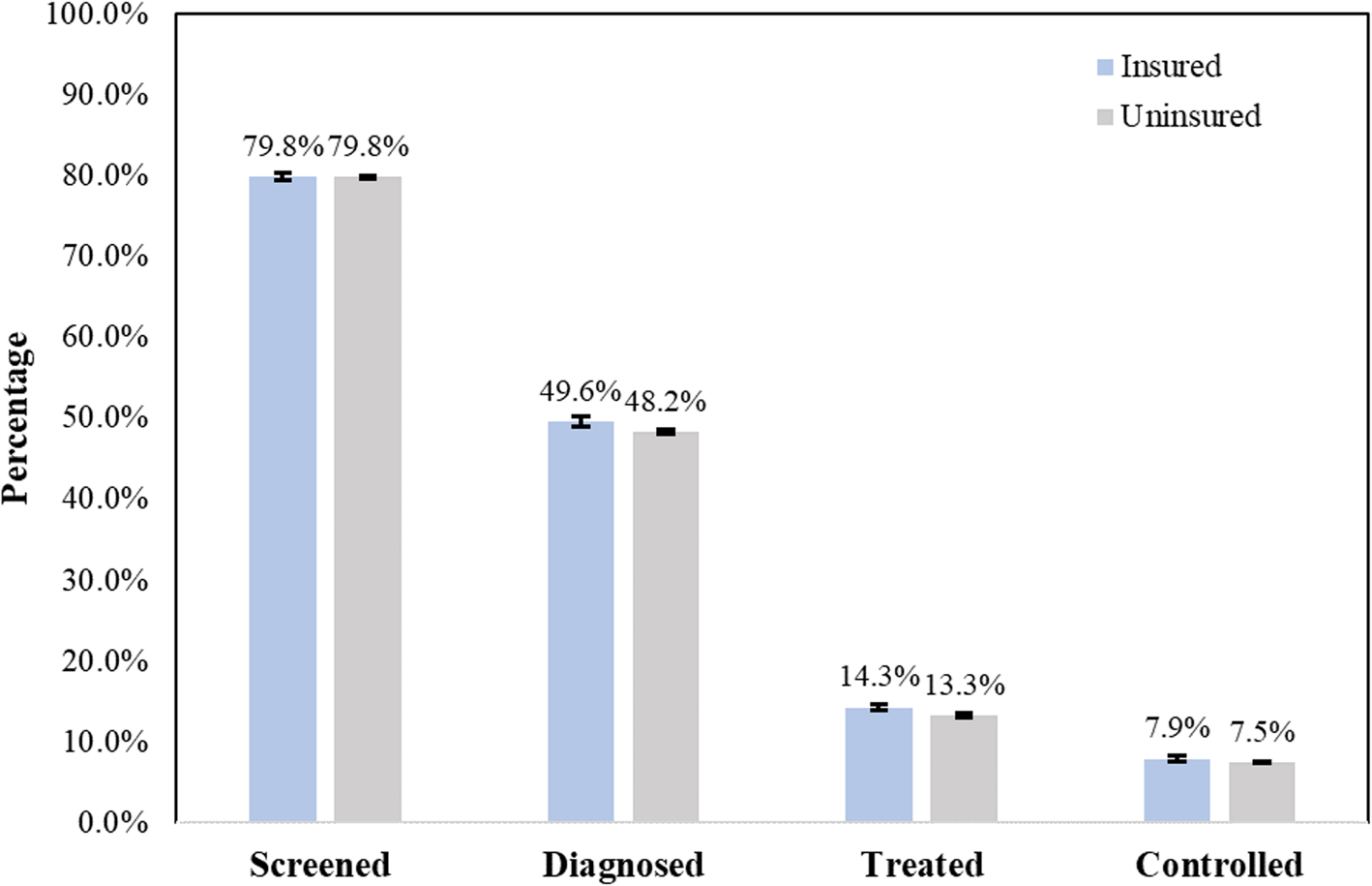
Proportion of hypertensive individuals reaching each cascade step by health insurance status

We identified 130,151 individuals with hypertension in our study cohort, among whom 26,568 (20.4%) were insured and 103,583 (79.6%) were uninsured. The prevalence of hypertension was estimated to be 19.4% (unweighted, 95% CI: 19.2%-19.6%) among the insured and 16.9% (95% CI: 16.9%-17.0%) among the uninsured. Figure 1 shows the unweighted proportions of hypertensive individuals reaching each cascade step by insurance status. Among insured individuals with hypertension, 79.8% (95% CI: 79.3%-80.3%) had been *screened*, 49.6% (95% CI: 49.0%-50.2%) had been *diagnosed*, 14.3% (95% CI: 13.9%-14.7%) had been *treated* with antihypertensive medications, and 7.9% (95% CI: 7.6%-8.2%) had *controlled* blood pressure; in comparison, among those without health insurance coverage, the respective proportions were estimated to be 79.8% (95% CI: 79.5%-80.0%, *p* = 0.87), 48.2% (95% CI: 47.9%-48.6%, *p* < 0.01), 13.3% (95% CI: 13.1%-13.5%, *p* < 0.01), and 7.5% (95% CI: 7.4%-7.7%, *p* < 0.01). Weighted estimates for cohort characteristics and care cascade outcomes by insurance coverage can be found in Supplementary Tables S1 and Figures S2.

The results of our household fixed effects models are summarized in Table 2. After adjusting for sex, age group, marital status, education level, BMI, tobacco consumption, and household fixed effects, we found no significant impacts of health insurance coverage on the likelihood of reaching each successive hypertension care cascade step of *screened, diagnosed, treated,* and *controlled*. Specifically, the estimated RRs for insured hypertensive individuals to uninsured hypertensive individuals were 0.97 (95% CI: 0.93–1.02) for reaching *screened*, 0.97 (95% CI: 0.91–1.03) for reaching *diagnosed*, 0.95 (95% CI: 0.77–1.30) for reaching *treated*, and 0.97 (95% CI: 0.65–1.10) for reaching *controlled*. Regression analysis conducted without applying sample weights yielded similar results (Table S2).

**Table 2:** Results of household fixed effects regression models for the impact of insurance coverage on the likelihood of reaching successive hypertension care cascade steps

	**Screened**		**Diagnosed**		**Treated**		**Controlled**	
	**RR** **(95% CI)**	***P*-value**	**RR** **(95% CI)**	***P*-value**	**RR** **(95% CI)**	***P*-value**	**RR** **(95% CI)**	***P*-value**
**Insurance Coverage**								
Uninsured	**1** **(reference)**		**1 (reference)**		**1 (reference)**		**1 (reference)**	
Insured	0.97 (0.93, 1.02)	0.25	0.97 (0.91, 1.03)	0.28	1 (0.77, 1.30)	0.98	0.85 (0.65, 1.10)	0.22
**Sex**								
Female	**1** **(reference)**		**1 (reference)**		**1 (reference)**		**1 (reference)**	
Male	0.93 (0.90, 0.96)	< 0.01	0.92 (0.87, 0.96)	< 0.01	0.95 (0.78, 1.16)	0.59	0.88 (0.64, 1.19)	0.40
**Age Group**								
15–19 years	**1** **(reference)**		**1 (reference)**		**1 (reference)**		**1 (reference)**	
20–24 years	1.07 (1.00, 1.14)	0.06	0.98 (0.94, 1.03)	0.49	0.65 (0.47, 0.90)	0.01	1.06 (0.85, 1.32)	0.59
25–29 years	1.16 (1.07, 1.25)	< 0.01	0.95 (0.90, 1.01)	0.09	0.79 (0.52, 1.19)	0.26	0.80 (0.57, 1.12)	0.20
30–34 years	1.14 (1.05, 1.24)	< 0.01	1.01 (0.93, 1.10)	0.76	0.89 (0.61, 1.30)	0.54	0.81 (0.57, 1.14)	0.23
35–39 years	1.17 (1.08, 1.26)	< 0.01	0.92 (0.86, 0.99)	0.02	0.75 (0.50, 1.14)	0.18	0.82 (0.56, 1.21)	0.31
40–44 years	1.19 (1.10, 1.30)	< 0.01	0.95 (0.88, 1.02)	0.18	1.14 (0.81, 1.61)	0.46	0.58 (0.38, 0.87)	0.01
45–49 years	1.17 (1.09, 1.26)	< 0.01	1.00 (0.95, 1.05)	0.97	1.28 (0.90, 1.82)	0.17	0.72 (0.49, 1.04)	0.08
**Marital Status**								
Unmarried	**1** **(reference)**		**1 (reference)**		**1 (reference)**		**1 (reference)**	
Married	1.15 (1.09, 1.21)	< 0.01	1.03 (0.99, 1.08)	0.18	1.23 (0.98, 1.54)	0.08	0.96 (0.75, 1.24)	0.78
**Education Level**								
No education	**1** **(reference)**		**1 (reference)**		**1 (reference)**		**1 (reference)**	
Primary school unfinished	0.99 (0.91, 1.07)	0.77	0.98 (0.87, 1.1)	0.72	0.46 (0.31, 0.67)	< 0.01	0.71 (0.49, 1.04)	0.08
Primary school finished	0.99 (0.94, 1.04)	0.66	1.00 (0.95, 1.06)	0.89	1.02 (0.72, 1.44)	0.92	1.14 (0.62, 2.07)	0.68
Secondary school unfinished	1.05 (1.01, 1.10)	0.03	1.03 (0.98, 1.08)	0.31	0.72 (0.54, 0.95)	0.02	1.11 (0.82, 1.50)	0.51
Secondary school finished	1.05 (0.98, 1.11)	0.14	0.99 (0.93, 1.05)	0.75	0.73 (0.52, 1.02)	0.06	0.88 (0.57, 1.36)	0.56
Secondary school above	1.10 (1.03, 1.17)	< 0.01	1.02 (0.96, 1.09)	0.47	0.60 (0.41, 0.86)	0.01	0.66 (0.39, 1.14)	0.14
**BMI**								
< 18.5 kg/m^2^ (Thin)	1.07 (1.03, 1.12)	< 0.01	1.04 (1.00, 1.08)	0.05	0.99 (0.76, 1.3)	0.96	0.91 (0.72, 1.16)	0.45
18.5–24.9 kg/m^2^ (Normal)	**1** **(reference)**		**1 (reference)**		**1 (reference)**		**1 (reference)**	
25.0–29.9 kg/m^2^ (Overweight)	0.98 (0.94, 1.02)	0.37	1.02 (0.98, 1.07)	0.38	1.12 (0.89, 1.42)	0.33	0.67 (0.50, 0.89)	0.01
> 30.0 kg/m^2^ (Obese)	1.1 (1.04, 1.17)	< 0.01	1.07 (0.98, 1.16)	0.13	1.15 (0.90, 1.47)	0.26	0.62 (0.39, 0.98)	0.04
**Smoking**								
Non-smoker	**1** **(reference)**		**1 (reference)**		**1 (reference)**		**1 (reference)**	
Current smoker	0.90 (0.83, 0.96)	< 0.01	1.04 (0.98, 1.10)	0.26	1.06 (0.73, 1.54)	0.76	0.57 (0.31, 1.06)	0.08
**Use of smokeless tobacco**								
Does not use	**1** **(reference)**		**1 (reference)**		**1 (reference)**		**1 (reference)**	
Uses smokeless tobacco	1.01 (0.94, 1.09)	0.75	0.96 (0.89, 1.05)	0.4	1.07 (0.67, 1.69)	0.78	1.26 (0.61, 2.58)	0.53

*RR* Relative risk, *95% CI* 95% confidence interval, *BMI* Body mass index

Our stratified analyses by sex, age groups, education levels, BMI categories, and smoking behaviors also showed a non-significant impact of health insurance coverage on the likelihood of reaching successive hypertension care cascade steps for all subgroups, except for the observation of modest impacts for *treated* and *controlled* among females and for *screened* among non-smokers (Tables S3-7). We further stratified the household fixed effects model by the residence (urban vs. rural) and household wealth categories (Tables S8-11). The results showed that health insurance does not significantly impact hypertension care for patients in the lower tertile (the poorest) household in both rural and urban areas of India. Indeed, across all stratified subgroups, no significant impacts were observed on the likelihood of reaching each hypertension care cascade step, except for a modest impact on the likelihood of being *treated* among individuals from middle wealth household residing in urban areas.

### Sensitivity analysis

Additional analyses using district-level fixed effects models were performed to include the household-level variables of urban vs. rural residence and wealth category as additional covariates (Table S12). These analyses showed a modest impact of insurance coverage on improving the likelihood of reaching the *screened* cascade step (RR = 1.02, 95% CI: 1.00–1.04), while the impacts on other care cascade steps (i.e., *diagnosed*, *treated*, and *controlled*) were not significant. These results further validated our base case findings.

## Discussion

In this study, we examined the impact of health insurance coverage on the likelihood of reaching the successive steps of screened, diagnosed, treated, and controlled in the hypertension care cascade among hypertensive individuals aged 15–49 years in India using a nationally representative cohort data from the 2015–2016 National Family Health Survey. While the aggregated estimates for the proportion of individuals reaching the cascade steps of *diagnosed*, *treated*, and *controlled* were significantly higher among those with health insurance coverage than those without insurance coverage, our analysis with household fixed effects models did not show significant impacts of self-reported health insurance status on the likelihood of reaching each step of the hypertension care cascade. Our sensitivity analyses, including stratified analyses by urban vs. rural residence and household wealth category and district-level fixed effect models, yielded results that agreed with our base case findings.

The non-significant relationship between health insurance coverage and the likelihood of reaching each successive step of the hypertension care cascade in our analysis may be attributable to several factors. In addition to health insurance coverage, the impact of health insurance on hypertension care outcomes is also likely to be influenced by what health services are included and utilized. During 2007–2010, several public health insurance schemes, such as Rashtriya Swasthiya Bima Yojna, Rajiv Aarogyasri scheme, and Rajiv Gandhi Jeevandayee Arogya Yojna, were launched at both the national and state levels in India; however, the coverage and benefits of these insurance schemes varied [43]. Moreover, the implementation and utilization of these schemes also varied across states despite similar program designs [44]. Most insurance programs do not include outpatient care expenses or medication costs for chronic diseases [45, 46] despite the financial burden that outpatient care imposes on impoverished households [44, 47]. Because hypertension management typically requires a series of outpatient follow-up visits [48], non-coverage of outpatient care costs could contribute to the lack of observed significance in the hypothesized impact of health insurance coverage on the likelihood of reaching successive steps of the hypertension care cascade. Furthermore, the enrollment rate, enrolled family sizes, hospital empanelment, and utilization under the same public health insurance scheme are found to differ significantly among different states [44, 49]. These variations could severely affect the nature of hypertension care received by individuals covered by health insurance. To address the above-mentioned challenges, policies and interventions aiming at reducing geographic disparities in healthcare service access under public health insurance schemes could be implemented to improve access to healthcare facilities and services [50], which is a key component of hypertension management [51].

The non-significant findings may also result from the longitudinal nature of hypertension care. Since this study did not follow patients over time as in a longitudinal study, we were unable to capture any potential lagged impacts of health insurance on hypertension care cascade outcomes. In our stratified analysis, insurance coverage showed modest effects on the *screened* cascade step in some subgroups. It may be easier to observe the effects of insurance on an early step of the hypertension care cascade because there are not as many confounders as for later cascade steps. Additional statistical analyses that can capture time lags are needed to provide more insight into the impact of health insurance on the hypertension care cascade.

Our study yielded similar findings to those from a recent similar study, despite that it focused on an older population (aged ≥ 45 years) in India [27]. The prior study found that among this older population in which hypertension is more prevalent, having health insurance was not related to improved hypertension awareness among the poor and middle economic classes, and was also not associated with an increased likelihood of reaching successive cascade steps (i.e., receiving treatment and controlling blood pressure) among all economic classes. The lack of significance in these results highlights that current health insurance coverage may not be sufficient for ensuring access to healthcare for hypertensive patients in India, especially for those among the poor and middle economic subgroups. Our study adds to the call for increasing equity by tailoring health insurance to be more inclusive of the poor, and we further stress the importance of improving access to healthcare, in particular within the health insurance context. On the other hand, unlike in this prior study, we did not observe a significant impact of insurance coverage on awareness among the rich subgroup, which implies that the impact of insurance could be more limited among the younger hypertensive population than it is among older age groups.

As hypertension becomes increasingly prevalent in children and young adults [52, 53], it is of paramount importance to promote access to preventive care services targeted at this population. Because risk factors for hypertension—for example, physical inactivity and poor diet—early in life may carry over to later adulthood [54, 55], proper preventive care could lead to long-term health benefits at both the individual and population levels. Strategies that have been promoted previously among the older population should be expanded to include young populations; these strategies include improving awareness of hypertension through health education programs [52, 55], early screening for blood pressure elevation [56], prescribing physical activity, active dietary counseling, and weight management programs [56, 57].

While expanding insurance coverage is important to increase access to affordable healthcare services and reduce financial barriers, the demand-side interventions that lead to the “entitlement” of insurance coverage are not enough to achieve the ultimate goal of improving health outcomes—as shown by our analysis as an example—which should be supplemented by supply-side interventions. The supply-side interventions, for example, include increasing the availability and accessibility of primary healthcare services, which are essential to address the gaps in healthcare delivery, particularly in rural and remote areas where access to healthcare is limited [58]. Furthermore, targeted policies are needed to reach at-risk populations, particularly those living in poverty, to ensure effective healthcare delivery and access to care.

To address the large unmet need for hypertension care in India among both resource-poor and resource-rich populations, policymakers and stakeholders may consider other innovative and contemporary approaches that can complement the ongoing efforts. For example, social media hypertension literacy interventions can leverage the power of social media platforms to increase awareness about hypertension and the related unhealthy lifestyle behaviors, such as physical inactivity and poor diet, which could lead to long-term health benefits at both individual and population levels [59]. Public hypertension screening campaigns have also been shown to increase the early detection and diagnosis of hypertension, which is critical for effectively managing and controlling this chronic disease [60–63]. These campaign programs can target high-risk populations or low-income households, which are also easy to be integrated into existing healthcare programs and services. In addition, community-based hypertension care, which involves engaging local communities and healthcare providers in the delivery of hypertension care, promoting patient self-management, and providing education and support for patients, and their families, is also effective approach, which has been shown to improve access to healthcare services and support effective hypertension management and control [64, 65].

Our study has several limitations. First, the survey dataset used in this study only included the population aged 15–49 years. Our findings of a non-significant impact of insurance coverage should not be directly extrapolated to the general population in India, especially for the older population who represent the major at-risk group for hypertension. Second, since the observations from the survey data were limited to a single visit, we were not able to evaluate the longitudinal effects of insurance coverage (such as by different durations of coverage) on long-term hypertension health outcomes. Third, given the variables collected by the survey, we defined the *treated* cascade step only in terms of adherence to antihypertension medications and did not account for other interventions on behavioral risk factors of hypertension, such as lifestyle modifications (e.g., increased physical exercise, reduced sodium intake, smoking cessation), that are also important and effective for maintaining blood pressure under control. Considering the importance of health behaviors on blood pressure control [66] and yet mixed evidence on the impact of health insurance coverage on health risk behaviors in general settings [67–70], future research is warranted to better understand the potential effects of health insurance on hypertension-related risk behaviors and its implications on the goal of achieving controlled blood pressure, which could also depend on the coverage of insurance and the context of the health system in India. Fourth, our analyses were based on NFHS-4 data that allowed us to employ a household fixed-effect study design, prior to the rollout of major public health insurance schemes with household-level insurance coverage. Given the changing landscape of health insurance policies and emerging data from more recent surveys, future research can further investigate how the health insurance policy changes impacted hypertension care cascade outcomes and identify the driving components in the insurance policy design for improving hypertension care. Lastly, due to the household fixed effects design, individuals from the households with only one member sampled did not contribute to the estimation in the regression model. To address this issue, we performed additional sensitivity analyses using district fixed effects models to utilize the full cohort, which yielded similar results and supported our main findings.

## Conclusions

Our study investigated the impact of health insurance coverage on hypertension care cascade outcomes among individuals aged 15–49 years in India using a nationally representative sample. Using household fixed effects models, we found that the overall impacts of self-reported health insurance coverage on reaching each step of the hypertension care cascade were not significant, despite the observations of a moderate positive impact on the *screened* step in several subgroups. Our results imply that improving financial access to healthcare through health insurance schemes, at least before the full roll-out of household-based insurance programs, may not substantially increase the proportion of individuals achieving blood pressure control among the young and middle-aged with hypertension in India. The lack of observed significant impacts of health insurance on hypertension care outcomes could be due to limitations to the services covered by insurance and other barriers to health service access in practice. Our findings suggest that having healthcare insurance—the coverage width—is a prerequisite but may not be sufficient for improving health outcomes. While expanding health insurance coverage width is an important strategy for achieving universal health coverage [71], it is also crucial to recognize the practical barriers to accessing healthcare services—the lack of coverage depth—that could also impede improvement in population health outcomes. Future research is needed to understand these barriers in the context of hypertension care. A better understanding would support health policymakers in designing effective health insurance programs and in improving healthcare provision in India and other contexts.

### Supplementary Information

Supplementary Material 1.

## Data Availability

The data underlying the results presented in the study are available from the Demographic and Health Surveys (DHS) Program at https://dhsprogram.com/data/dataset/India_Standard-DHS_2015.cfm?flag=0.
