# Supplementary material for: The impact of health insurance on hypertension care: a household fixed effects study in India
Source: BMC Public Health. 2024 Aug 22;24:2287. doi: 10.1186/s12889-024-19759-1 (PMC11342611; doi:10.1186/s12889-024-19759-1)
Supplement: Supplementary file 1 — Supplementary Material 1. [file 12889_2024_19759_MOESM1_ESM.docx]

**SUPPLEMENTAL MATERIALS**

**Figure S1.** Flowchart illustrating the cohort selection and the identification of the hypertensive population (unweighted)

Hypertensive population (n=130,151)

Identified by at least one of the following criteria:

♦ Having raised BP measurements (n=84,776)

♦ Having a previous diagnosis of hypertension (n=63,146)

Excluded (n=618,245)

♦  Without hypertension

Excluded (n=22,387)

♦  Abnormal BP measurements

(n= 16,756)

♦  Inconsistent or incomplete responses (n= 20,754)

Excluded (n=32,428)

♦  Pregnant females

Excluded (n=8,597)

♦  Aged <15 or >49 years old

Males and non-pregnant females (n=770,783)

Samples with valid responses (n=748,396)

Aged between 15-49 years old (n=803,211)

Sampled by NFHS-4

(n=811,808)

**Figure S2**. Weighted proportions of the hypertensive population reaching each hypertension care cascade step by self-reported health insurance status.

**Table S1.** Sample characteristics of the selected cohort from the 2015-2016 National Family Health Survey in India (weighted).

|  | **Overall** | **Insured** | **Uninsured** | **P-value^*^** |
| --- | --- | --- | --- | --- |
| **Number of individuals, N** | **744,367** | **155,543** | **588,824** |  |
| **Age (years)** |  |  |  | <0.01 |
| Mean (SD) | 30.2 (9.9) | 31.9 (10.0) | 29.8 (9.8) |  |
| Median [Min, Max] | 30.0 [15.0, 49.0] | 32.0 [15.0, 49.0] | 29.0 [15.0, 49.0] |  |
| **Age group** |  |  |  | <0.01 |
| 15-19 years | 131,243 (17.6%) | 22,702 (14.6%) | 108,541 (18.4%) |  |
| 20-24 years | 121,071 (16.3%) | 20,319 (13.1%) | 100,752 (17.1%) |  |
| 25-29 years | 117,632 (15.8%) | 22,059 (14.2%) | 95,573 (16.2%) |  |
| 30-34 years | 104,592 (14.1%) | 22,600 (14.5%) | 81,993 (13.9%) |  |
| 35-39 years | 100,270 (13.5%) | 24,248 (15.6%) | 76,021 (12.9%) |  |
| 40-44 years | 86,835 (11.7%) | 21,558 (13.9%) | 65,277 (11.1%) |  |
| 45-49 years | 82,724 (11.1%) | 22,057 (14.2%) | 60,667 (10.3%) |  |
| **Sex** |  |  |  | <0.01 |
| Male | 98,604 (13.2%) | 22,575 (14.5%) | 76,029 (12.9%) |  |
| Female | 645,763 (86.8%) | 132,968 (85.5%) | 512,795 (87.1%) |  |
| **Residence** |  |  |  | <0.01 |
| Urban | 258,519 (34.7%) | 51,505 (33.1%) | 207,014 (35.2%) |  |
| Rural | 485,848 (65.3%) | 104,038 (66.9%) | 381,810 (64.8%) |  |
| **Household wealth** |  |  |  | <0.01 |
| Lower | 239,187 (32.1%) | 43,015 (27.7%) | 196,173 (33.3%) |  |
| Middle | 250,342 (33.6%) | 56,405 (36.3%) | 193,938 (32.9%) |  |
| Upper | 254,837 (34.2%) | 56,123 (36.1%) | 198,714 (33.7%) |  |
| **Diastolic blood pressure (mmHg)** |  |  |  | <0.01 |
| Mean (SD) | 77.6 (12.2) | 78.0 (12.1) | 77.6 (12.2) |  |
| Median [Min, Max] | 76.7 [26.0, 299.7] | 77.0 [33.3, 299.7] | 76.7 [26.0, 299.7] |  |
| **Systolic blood pressure (mmHg)** |  |  |  | <0.01 |
| Mean (SD) | 115.42 (14.1) | 116.2 (14.6) | 115.4 (14.0) |  |
| Median [Min, Max] | 114.3 [34.3, 299.7] | 114.0 [47.7, 292.0] | 114.3 [34.3, 299.7] |  |
| **Education Level** |  |  |  | <0.01 |
| No education | 190,615 (25.6%) | 39,577 (25.4%) | 151,039 (25.7%) |  |
| Primary school unfinished | 44,965 (6.0%) | 10,754 (6.9%) | 34,211 (5.8%) |  |
| Primary school finished | 48,076 (6.5%) | 10,013 (6.4%) | 38,063 (6.5%) |  |
| Secondary school unfinished | 295,596 (39.7%) | 60,575 (38.9%) | 235,021 (39.9%) |  |
| Secondary school finished | 67,204 (9.0%) | 13,139 (8.4%) | 54,066 (9.2%) |  |
| Secondary school above | 97,910 (13.2%) | 21,486 (13.8%) | 76,424 (13.0%) |  |
| **Marital Status** |  |  |  | <0.01 |
| Unmarried | 219,295 (29.5%) | 42,676 (17.4%) | 176,619 (30.0%) |  |
| Married | 525,072 (70.5%) | 112,867 (72.6%) | 412,205 (70.0%) |  |
| **BMI** |  |  |  | <0.01 |
| <18.5 kg/m^2^ (Thin) | 424,532 (57.0%) | 87,098 (56.0%) | 337,435 (57.3%) |  |
| 18.5-24.9 kg/m^2^ (Normal) | 168,134 (22.6%) | 31,606 (20.3%) | 136,528 (23.2%) |  |
| 25.0-29.9 kg/m^2^ (Overweight) | 115,392 (15.5%) | 27,652 (17.8%) | 87,739 (14.9%) |  |
| >30.0 kg/m^2^ (Obese) | 36,308 (4.9%) | 9,187 (5.9%) | 27,125 (4.6%) |  |
| **Tobacco Consumption** |  |  |  |  |
| Current smoker | 89,319 (12.0%) | 20,397 (13.1%) | 68,921 (11.7%) | <0.01 |
| Uses smokeless tobacco | 65,970 (8.9%) | 13,555 (8.7%) | 52,415 (8.9%) | <0.01 |
| **Hypertension care** |  |  |  |  |
| Has hypertension | 128,679 (17.3%) | 31,123 (20.0%) | 97556 (16.6%) | <0.01 |
| Screened | 445,913 (59.9%) | 97,977 (63.0%) | 347,936 (59.1%) | <0.01 |
| Diagnosed | 65,099 (8.7%) | 16,838 (10.8%) | 48,262 (8.2%) | <0.01 |
| Treated | 18,104 (2.4%) | 4,757 (3.1%) | 13,348 (2.3%) | <0.01 |
| Controlled | 10,549 (1.4%) | 2,726 (1.8%) | 7,823 (1.3%) | <0.01 |

*Note*: Summary statistics in this table are weighted.

*** P-values were for the comparison of characteristics between insured and uninsured groups.

*Abbreviations*: SD, standard deviation; Min, minimum; Max, maximum; BMI, body mass index

**Table S2.** Results of household fixed effects regression models for the impact of insurance coverage on the likelihood of reaching successive hypertension care cascade steps without weights.

|  | **Screened** | | **Diagnosed** | | **Treated** | | **Controlled** | |
| --- | --- | --- | --- | --- | --- | --- | --- | --- |
|  | **RR**  **(95% CI)** | **P-value** | **RR**  **(95% CI)** | **P-value** | **RR**  **(95% CI)** | **P-value** | **RR**  **(95% CI)** | **P-value** |
| **Insurance Coverage** |  |  |  |  |  |  |  |  |
| Uninsured | **1 (reference)** |  | **1 (reference)** |  | **1 (reference)** |  | **1 (reference)** |  |
| Insured | 1.00  (0.98, 1.03) | 0.87 | 0.99  (0.96, 1.02) | 0.37 | 1.10  (0.94, 1.30) | 0.22 | 0.89  (0.77, 1.03) | 0.10 |
| **Sex** |  |  |  |  |  |  |  |  |
| Female | **1 (reference)** |  | **1 (reference)** |  | **1 (reference)** |  | **1 (reference)** |  |
| Male | 0.89  (0.87, 0.90) | <0.01 | 0.90  (0.88, 0.93) | <0.01 | 0.97  (0.84, 1.11) | 0.64 | 0.79  (0.66, 0.93) | 0.01 |
| **Age Group** |  |  |  |  |  |  |  |  |
| 15-19 years | **1 (reference)** |  | **1 (reference)** |  | **1 (reference)** |  | **1 (reference)** |  |
| 20-24 years | 1.07  (1.04, 1.10) | <0.01 | 0.98  (0.95, 1.00) | 0.09 | 0.75  (0.63, 0.90) | <0.01 | 1.08  (0.92, 1.28) | 0.35 |
| 25-29 years | 1.12  (1.08, 1.15) | <0.01 | 0.96  (0.93, 1.00) | 0.03 | 0.72  (0.58, 0.90) | <0.01 | 1.00  (0.82, 1.22) | 1.00 |
| 30-34 years | 1.14  (1.10, 1.18) | <0.01 | 0.98  (0.95, 1.02) | 0.36 | 0.98  (0.76, 1.25) | 0.85 | 0.96  (0.76, 1.22) | 0.75 |
| 35-39 years | 1.12  (1.08, 1.16) | <0.01 | 0.97  (0.93, 1.00) | 0.07 | 1.10  (0.88, 1.38) | 0.42 | 0.82  (0.65, 1.05) | 0.12 |
| 40-44 years | 1.13  (1.10, 1.17) | <0.01 | 0.96  (0.93, 1.00) | 0.03 | 1.22  (0.99, 1.50) | 0.06 | 0.77  (0.60, 0.97) | 0.03 |
| 45-49 years | 1.14  (1.10, 1.18) | <0.01 | 1.00  (0.96, 1.03) | 0.87 | 1.36  (1.11, 1.68) | <0.01 | 0.78  (0.61, 0.99) | 0.04 |
| **Marital Status** |  |  |  |  |  |  |  |  |
| Unmarried | **1 (reference)** |  | **1 (reference)** |  | **1 (reference)** |  | **1 (reference)** |  |
| Married | 1.14  (1.12, 1.16) | <0.01 | 1.03  (1.01, 1.06) | <0.01 | 1.07  (0.93, 1.24) | 0.34 | 0.99  (0.84, 1.17) | 0.94 |
| **Education Level** |  |  |  |  |  |  |  |  |
| No education | **1 (reference)** |  | **1 (reference)** |  | **1 (reference)** |  | **1 (reference)** |  |
| Primary school unfinished | 1.00  (0.96, 1.04) | 0.94 | 1.02  (0.97, 1.07) | 0.39 | 0.80  (0.64, 1.00) | 0.05 | 0.72  (0.55, 0.93) | 0.01 |
| Primary school finished | 1.00  (0.96, 1.03) | 0.86 | 0.99  (0.96, 1.03) | 0.75 | 0.96  (0.76, 1.20) | 0.71 | 1.02  (0.80, 1.31) | 0.85 |
| Secondary school unfinished | 1.00  (0.98, 1.03) | 0.79 | 1.00  (0.98, 1.03) | 0.78 | 0.92  (0.79, 1.07) | 0.26 | 0.97  (0.82, 1.14) | 0.72 |
| Secondary school finished | 1.03  (1.00, 1.06) | 0.08 | 1.00  (0.96, 1.03) | 0.83 | 0.89  (0.73, 1.08) | 0.23 | 0.93  (0.74, 1.16) | 0.51 |
| Secondary school above | 1.04  (1.01, 1.08) | 0.01 | 1.01  (0.98, 1.05) | 0.51 | 0.83  (0.67, 1.02) | 0.08 | 0.90  (0.71, 1.14) | 0.37 |
| **BMI** |  |  |  |  |  |  |  |  |
| <18.5 kg/m^2^ (Thin) | 1.01  (0.99, 1.04) | 0.31 | 1.03  (1.01, 1.06) | <0.01 | 1.00  (0.87, 1.16) | 0.98 | 1.13  (0.99, 1.29) | 0.08 |
| 18.5-24.9 kg/m^2^ (Normal) | **1 (reference)** |  | **1 (reference)** |  | **1 (reference)** |  | **1 (reference)** |  |
| 25.0-29.9 kg/m^2^ (Overweight) | 1.01  (0.99, 1.03) | 0.24 | 1.00  (0.98, 1.02) | 0.93 | 1.17  (1.04, 1.33) | 0.01 | 0.85  (0.73, 0.99) | 0.04 |
| >30.0 kg/m^2^ (Obese) | 1.05  (1.02, 1.08) | <0.01 | 1.04  (1.00, 1.08) | 0.04 | 1.27  (1.08, 1.49) | <0.01 | 1.00  (0.79, 1.28) | 0.98 |
| **Smoking** |  |  |  |  |  |  |  |  |
| Non-smoker | **1 (reference)** |  | **1 (reference)** |  | **1 (reference)** |  | **1 (reference)** |  |
| Current smoker | 0.99  (0.95, 1.02) | 0.39 | 1.01  (0.97, 1.05) | 0.57 | 0.97  (0.78, 1.20) | 0.75 | 0.81  (0.61, 1.08) | 0.15 |
| **Use of smokeless tobacco** |  |  |  |  |  |  |  |  |
| Does not use | **1 (reference)** |  | **1 (reference)** |  | **1 (reference)** |  | **1 (reference)** |  |
| Uses smokeless tobacco | 0.97  (0.93, 1.01) | 0.12 | 0.99  (0.94, 1.04) | 0.75 | 1.06  (0.83, 1.35) | 0.64 | 1.22  (0.86, 1.74) | 0.27 |
| Abbreviations: RR, relative risk; 95% CI, 95% confidence interval; BMI, body mass index. | | | | | | | | |

**Table S3.** Results of household fixed effects regression models for the impact of insurance coverage on the likelihood of reaching successive hypertension care cascade steps stratified by sex.

| **Sex: Male** | | | | | | | | |
| --- | --- | --- | --- | --- | --- | --- | --- | --- |
|  | **Screened** | | **Diagnosed** | | **Treated** | | **Controlled** | |
|  | **RR**  **(95% CI)** | **P-value** | **RR**  **(95% CI)** | **P-value** | **RR**  **(95% CI)** | **P-value** | **RR**  **(95% CI)** | **P-value** |
| **Insurance Coverage** |  |  |  |  |  |  |  |  |
| Uninsured | **1 (reference)** |  | **1 (reference)** |  | **1 (reference)** |  | **1 (reference)** |  |
| Insured | 0.87  (0.74, 1.03) | 0.10 | 0.91  (0.76, 1.1) | 0.35 | 0.66  (0.24, 1.77) | 0.40 | 1.55  (0.42, 5.70) | 0.51 |
| **Age Group** |  |  |  |  |  |  |  |  |
| 15-19 years | **1 (reference)** |  | **1 (reference)** |  | **1 (reference)** |  | **1 (reference)** |  |
| 20-24 years | 1.09  (0.93, 1.28) | 0.27 | 0.99  (0.88, 1.10) | 0.80 | 0.48  (0.19, 1.17) | 0.11 | 0.46  (0.14, 1.47) | 0.19 |
| 25-29 years | 1.22  (0.99, 1.50) | 0.07 | 0.98  (0.83, 1.16) | 0.85 | 1.25  (0.41, 3.82) | 0.70 | 0.71  (0.09, 5.65) | 0.75 |
| 30-34 years | 1.17  (0.94, 1.47) | 0.16 | 1.18  (0.87, 1.59) | 0.28 | 0.69  (0.12, 3.95) | 0.68 | 0.67 (0.04, 10.70) | 0.78 |
| 35-39 years | 1.24  (0.99, 1.56) | 0.06 | 0.88  (0.65, 1.19) | 0.40 | 0.40  (0.05, 3.13) | 0.38 | 1.17 (0.02, 73.83) | 0.94 |
| 40-44 years | 1.45  (1.14, 1.85) | <0.01 | 0.84  (0.64, 1.10) | 0.20 | 1.53  (0.37, 6.28) | 0.55 | 5.45 (0.17, 178.53) | 0.34 |
| 45-49 years | 1.27  (1.06, 1.52) | 0.01 | 0.99  (0.86, 1.15) | 0.92 | 1.57  (0.52, 4.72) | 0.43 | 2.01 (0.22, 18.73) | 0.54 |
| **Marital Status** |  |  |  |  |  |  |  |  |
| Unmarried | **1 (reference)** |  | **1 (reference)** |  | **1 (reference)** |  | **1 (reference)** |  |
| Married | 1.18  (1.04, 1.34) | 0.01 | 1.02  (0.89, 1.17) | 0.78 | 1.26  (0.49, 3.27) | 0.63 | 0.08  (0.01, 0.69) | 0.02 |
| **Education Level** |  |  |  |  |  |  |  |  |
| No education | **1 (reference)** |  | **1 (reference)** |  | **1 (reference)** |  | **1 (reference)** |  |
| Primary school unfinished | 0.94  (0.72, 1.23) | 0.66 | 0.97  (0.72, 1.30) | 0.84 | 0.10  (0.01, 1.02) | 0.05 | 5.99 (0.84, 42.57) | 0.07 |
| Primary school finished | 0.89  (0.70, 1.15) | 0.38 | 0.96  (0.75, 1.23) | 0.75 | 0.98  (0.10, 9.57) | 0.98 | 27.74 (0.44, 1749.39) | 0.12 |
| Secondary school unfinished | 1.12  (0.95, 1.33) | 0.19 | 1.09  (0.90, 1.32) | 0.39 | 0.35  (0.07, 1.77) | 0.20 | 13.35 (0.87, 205.25) | 0.06 |
| Secondary school finished | 1.13  (0.92, 1.38) | 0.24 | 0.98  (0.77, 1.25) | 0.90 | 0.23  (0.04, 1.43) | 0.11 | 2.8  (0.18, 43.53) | 0.46 |
| Secondary school above | 1.19  (0.96, 1.48) | 0.12 | 1.06  (0.84, 1.33) | 0.62 | 0.16  (0.02, 1.07) | 0.06 | 0.42  (0.03, 6.33) | 0.53 |
| **BMI** |  |  |  |  |  |  |  |  |
| <18.5 kg/m^2^ (Thin) | 1.19  (1.04, 1.36) | 0.01 | 1.05  (0.92, 1.19) | 0.48 | 1.00  (0.41, 2.45) | 1.00 | 0.41  (0.12, 1.35) | 0.14 |
| 18.5-24.9 kg/m^2^ (Normal) | **1 (reference)** |  | **1 (reference)** |  | **1 (reference)** |  | **1 (reference)** |  |
| 25.0-29.9 kg/m^2^ (Overweight) | 0.96  (0.86, 1.08) | 0.50 | 1.07  (0.94, 1.21) | 0.31 | 1.40  (0.77, 2.56) | 0.27 | 1.09  (0.81, 1.47) | 0.55 |
| >30.0 kg/m^2^ (Obese) | 1.21  (0.98, 1.51) | 0.08 | 1.08  (0.82, 1.42) | 0.57 | 1.34  (0.54, 3.35) | 0.53 | NA | NA |
| **Smoking** |  |  |  |  |  |  |  |  |
| Non-smoker | **1 (reference)** |  | **1 (reference)** |  | **1 (reference)** |  | **1 (reference)** |  |
| Current smoker | 0.79  (0.68, 0.92) | <0.01 | 1.07  (0.92, 1.23) | 0.38 | 0.93  (0.38, 2.27) | 0.87 | 0.54 (0.03, 10.00) | 0.68 |
| **Use of smokeless tobacco** |  |  |  |  |  |  |  |  |
| Does not use | **1 (reference)** |  | **1 (reference)** |  | **1 (reference)** |  | **1 (reference)** |  |
| Uses smokeless tobacco | 1.10  (0.95, 1.27) | 0.19 | 0.97  (0.83, 1.14) | 0.70 | 1.24  (0.47, 3.28) | 0.67 | 1.03  (0.11, 9.73) | 0.98 |
| **Sex: Female** | | | | | | | | |
|  | **Screened** | | **Diagnosed** | | **Treated** | | **Controlled** | |
|  | **RR**  **(95% CI)** | **P-value** | **RR**  **(95% CI)** | **P-value** | **RR**  **(95% CI)** | **P-value** | **RR**  **(95% CI)** | **P-value** |
| **Insurance Coverage** |  |  |  |  |  |  |  |  |
| Uninsured | **1 (reference)** |  | **1 (reference)** |  | **1 (reference)** |  | **1 (reference)** |  |
| Insured | 1.01  (0.96, 1.06) | 0.66 | 0.98  (0.94, 1.02) | 0.24 | 1.50  (1.10, 2.04) | 0.01 | 0.78  (0.63, 0.97) | 0.02 |
| **Age Group** |  |  |  |  |  |  |  |  |
| 15-19 years | **1 (reference)** |  | **1 (reference)** |  | **1 (reference)** |  | **1 (reference)** |  |
| 20-24 years | 1.05  (1.02, 1.09) | <0.01 | 1.00  (0.97, 1.03) | 0.99 | 0.68  (0.51, 0.90) | 0.01 | 1.24  (0.99, 1.55) | 0.07 |
| 25-29 years | 1.09  (1.04, 1.14) | <0.01 | 0.96  (0.92, 1.00) | 0.04 | 0.70  (0.51, 0.97) | 0.03 | 1.02  (0.81, 1.29) | 0.85 |
| 30-34 years | 1.09  (1.04, 1.14) | <0.01 | 0.98  (0.93, 1.03) | 0.35 | 0.88  (0.60, 1.28) | 0.50 | 0.88  (0.66, 1.16) | 0.37 |
| 35-39 years | 1.08  (1.03, 1.13) | <0.01 | 0.96  (0.92, 1.00) | 0.07 | 0.90  (0.61, 1.33) | 0.61 | 0.82  (0.59, 1.14) | 0.24 |
| 40-44 years | 1.10  (1.05, 1.16) | <0.01 | 1.01  (0.97, 1.06) | 0.68 | 1.12  (0.81, 1.53) | 0.50 | 0.77  (0.56, 1.06) | 0.11 |
| 45-49 years | 1.07  (1.02, 1.12) | 0.01 | 0.99  (0.95, 1.04) | 0.72 | 1.19  (0.83, 1.72) | 0.34 | 0.79  (0.58, 1.07) | 0.13 |
| **Marital Status** |  |  |  |  |  |  |  |  |
| Unmarried | **1 (reference)** |  | **1 (reference)** |  | **1 (reference)** |  | **1 (reference)** |  |
| Married | 1.12  (1.09, 1.15) | <0.01 | 1.04  (1.01, 1.06) | 0.01 | 1.27  (1.01, 1.60) | 0.04 | 1.09  (0.87, 1.36) | 0.45 |
| **Education Level** |  |  |  |  |  |  |  |  |
| No education | **1 (reference)** |  | **1 (reference)** |  | **1 (reference)** |  | **1 (reference)** |  |
| Primary school unfinished | 0.99  (0.93, 1.06) | 0.87 | 1.07  (0.99, 1.16) | 0.11 | 0.65  (0.44, 0.97) | 0.03 | 0.68  (0.45, 1.03) | 0.07 |
| Primary school finished | 0.98  (0.94, 1.02) | 0.33 | 0.98  (0.92, 1.03) | 0.38 | 0.86  (0.62, 1.21) | 0.40 | 0.88  (0.61, 1.28) | 0.50 |
| Secondary school unfinished | 1.00  (0.97, 1.04) | 0.98 | 1.00  (0.96, 1.04) | 0.92 | 0.68  (0.51, 0.92) | 0.01 | 1.16  (0.91, 1.48) | 0.24 |
| Secondary school finished | 1.01  (0.96, 1.05) | 0.82 | 0.99  (0.95, 1.04) | 0.75 | 0.72  (0.51, 1.02) | 0.06 | 0.99  (0.71, 1.36) | 0.93 |
| Secondary school above | 1.02  (0.97, 1.06) | 0.48 | 1.01  (0.96, 1.07) | 0.72 | 0.76  (0.52, 1.10) | 0.14 | 1.18  (0.84, 1.65) | 0.35 |
| **BMI** |  |  |  |  |  |  |  |  |
| <18.5 kg/m^2^ (Thin) | 1.03  (1.00, 1.06) | 0.03 | 1.03  (1.01, 1.06) | 0.01 | 1.07  (0.86, 1.33) | 0.53 | 1.09  (0.91, 1.29) | 0.35 |
| 18.5-24.9 kg/m^2^ (Normal) | **1 (reference)** |  | **1 (reference)** |  | **1 (reference)** |  | **1 (reference)** |  |
| 25.0-29.9 kg/m^2^ (Overweight) | 1.02  (0.99, 1.04) | 0.24 | 0.99  (0.96, 1.02) | 0.51 | 1.13  (0.88, 1.46) | 0.34 | 0.68  (0.55, 0.84) | <0.01 |
| >30.0 kg/m^2^ (Obese) | 1.02  (0.98, 1.07) | 0.30 | 1.00  (0.93, 1.07) | 0.90 | 1.15  (0.86, 1.53) | 0.35 | 0.99  (0.71, 1.37) | 0.95 |
| **Smoking** |  |  |  |  |  |  |  |  |
| Non-smoker | **1 (reference)** |  | **1 (reference)** |  | **1 (reference)** |  | **1 (reference)** |  |
| Current smoker | 1.05  (0.91, 1.22) | 0.51 | 0.96  (0.87, 1.07) | 0.48 | 1.37  (0.71, 2.68) | 0.35 | 1.08  (0.65, 1.80) | 0.77 |
| **Use of smokeless tobacco** |  |  |  |  |  |  |  |  |
| Does not use | **1 (reference)** |  | **1 (reference)** |  | **1 (reference)** |  | **1 (reference)** |  |
| Uses smokeless tobacco | 0.93  (0.79, 1.10) | 0.40 | 1.01  (0.90, 1.14) | 0.85 | 0.52  (0.25, 1.10) | 0.09 | 0.94  (0.53, 1.66) | 0.82 |
| Abbreviations: RR, relative risk; 95% CI, 95% confidence interval; BMI, body mass index. | | | | | | | | |

**Table S4**. Results of household fixed effects regression models for the impact of insurance coverage on the likelihood of reaching successive hypertension care cascade steps stratified by age groups.

| **Age group: >30 years** | | | | | | | | |
| --- | --- | --- | --- | --- | --- | --- | --- | --- |
|  | **Screened** | | **Diagnosed** | | **Treated** | | **Controlled** | |
|  | **RR**  **(95% CI)** | **P-value** | **RR**  **(95% CI)** | **P-value** | **RR**  **(95% CI)** | **P-value** | **RR**  **(95% CI)** | **P-value** |
| **Insurance Coverage** |  |  |  |  |  |  |  |  |
| Uninsured | **1 (reference)** |  | **1 (reference)** |  | **1 (reference)** |  | **1 (reference)** |  |
| Insured | 1.04  (0.98, 1.1) | 0.26 | 0.91  (0.81, 1.03) | 0.14 | 1.05  (0.73, 1.52) | 0.79 | 0.82  (0.38, 1.74) | 0.60 |
| **Sex** |  |  |  |  |  |  |  |  |
| Female | **1 (reference)** |  | **1 (reference)** |  | **1 (reference)** |  | **1 (reference)** |  |
| Male | 0.92  (0.89, 0.96) | <0.01 | 0.92  (0.85, 1.00) | 0.04 | 1.32  (0.99, 1.76) | 0.05 | 0.76  (0.52, 1.12) | 0.16 |
| **Marital Status** |  |  |  |  |  |  |  |  |
| Unmarried | **1 (reference)** |  | **1 (reference)** |  | **1 (reference)** |  | **1 (reference)** |  |
| Married | 1.09  (0.98, 1.20) | 0.11 | 0.98  (0.83, 1.15) | 0.77 | 1.03  (0.62, 1.69) | 0.91 | 0.62  (0.25, 1.56) | 0.31 |
| **Education Level** |  |  |  |  |  |  |  |  |
| No education | **1 (reference)** |  | **1 (reference)** |  | **1 (reference)** |  | **1 (reference)** |  |
| Primary school unfinished | 1.06  (0.94, 1.18) | 0.33 | 0.88  (0.67, 1.15) | 0.34 | 0.57  (0.31, 1.05) | 0.07 | 0.62  (0.19, 2.00) | 0.43 |
| Primary school finished | 1.04  (0.94, 1.15) | 0.41 | 1.03  (0.94, 1.12) | 0.53 | 1.09  (0.68, 1.73) | 0.72 | 1.25  (0.31, 5.05) | 0.75 |
| Secondary school unfinished | 1.05  (0.97, 1.14) | 0.25 | 1.02  (0.92, 1.13) | 0.73 | 0.75  (0.50, 1.12) | 0.16 | 0.88  (0.42, 1.86) | 0.75 |
| Secondary school finished | 0.99  (0.88, 1.11) | 0.88 | 1.01  (0.88, 1.16) | 0.88 | 0.76  (0.40, 1.43) | 0.39 | 0.92  (0.28, 2.96) | 0.89 |
| Secondary school above | 1.04  (0.93, 1.16) | 0.53 | 1.07  (0.93, 1.25) | 0.34 | 0.68  (0.39, 1.19) | 0.18 | 0.58  (0.23, 1.47) | 0.25 |
| **BMI** |  |  |  |  |  |  |  |  |
| <18.5 kg/m^2^ (Thin) | 1.03  (0.95, 1.11) | 0.49 | 0.99  (0.89, 1.09) | 0.80 | 0.75  (0.39, 1.43) | 0.39 | 1.74  (0.76, 3.99) | 0.19 |
| 18.5-24.9 kg/m^2^ (Normal) | **1 (reference)** |  | **1 (reference)** |  | **1 (reference)** |  | **1 (reference)** |  |
| 25.0-29.9 kg/m^2^ (Overweight) | 1.02  (0.96, 1.07) | 0.57 | 1.05  (0.98, 1.14) | 0.19 | 1.29  (0.95, 1.75) | 0.11 | 1.00  (0.52, 1.93) | 0.99 |
| >30.0 kg/m^2^ (Obese) | 1.12  (1.04, 1.21) | <0.01 | 1.11  (0.96, 1.29) | 0.17 | 1.21  (0.87, 1.67) | 0.25 | 0.84  (0.42, 1.67) | 0.61 |
| **Smoking** |  |  |  |  |  |  |  |  |
| Non-smoker | **1 (reference)** |  | **1 (reference)** |  | **1 (reference)** |  | **1 (reference)** |  |
| Current smoker | 0.95  (0.88, 1.02) | 0.17 | 1.10  (0.98, 1.24) | 0.10 | 0.77  (0.52, 1.15) | 0.20 | 0.51  (0.21, 1.26) | 0.15 |
| **Use of smokeless tobacco** |  |  |  |  |  |  |  |  |
| Does not use | **1 (reference)** |  | **1 (reference)** |  | **1 (reference)** |  | **1 (reference)** |  |
| Uses smokeless tobacco | 1.00  (0.91, 1.10) | 0.98 | 0.89  (0.79, 0.99) | 0.04 | 1.17  (0.76, 1.80) | 0.47 | 2.25  (0.88, 5.76) | 0.09 |
| **Age group: ≤30 years** | | | | | | | | |
|  | **Screened** | | **Diagnosed** | | **Treated** | | **Controlled** | |
|  | **RR** | **P-value** | **RR** | **P-value** | **RR** | **P-value** | **RR** | **P-value** |
| **Insurance Coverage** |  |  |  |  |  |  |  |  |
| Uninsured | **1 (reference)** |  | **1 (reference)** |  | **1 (reference)** |  | **1 (reference)** |  |
| Insured | 0.96  (0.85, 1.07) | 0.44 | 0.98  (0.92, 1.04) | 0.49 | 1.34  (0.79, 2.28) | 0.28 | 0.95  (0.83, 1.07) | 0.39 |
| **Sex** |  |  |  |  |  |  |  |  |
| Female | **1 (reference)** |  | **1 (reference)** |  | **1 (reference)** |  | **1 (reference)** |  |
| Male | 0.95  (0.88, 1.01) | 0.11 | 0.93  (0.9, 0.97) | <0.01 | 0.80  (0.37, 1.71) | 0.57 | 0.81  (0.46, 1.43) | 0.47 |
| **Marital Status** |  |  |  |  |  |  |  |  |
| Unmarried | **1 (reference)** |  | **1 (reference)** |  | **1 (reference)** |  | **1 (reference)** |  |
| Married | 1.24  (1.11, 1.38) | <0.01 | 1.00  (0.93, 1.07) | 0.91 | 0.99  (0.66, 1.46) | 0.94 | 0.60  (0.33, 1.10) | 0.10 |
| **Education Level** |  |  |  |  |  |  |  |  |
| No education | **1 (reference)** |  | **1 (reference)** |  | **1 (reference)** |  | **1 (reference)** |  |
| Primary school unfinished | 1.03  (0.86, 1.24) | 0.72 | 0.83  (0.65, 1.06) | 0.13 | 0.66  (0.30, 1.47) | 0.31 | 1.14  (0.67, 1.94) | 0.63 |
| Primary school finished | 0.90  (0.77, 1.06) | 0.20 | 0.96  (0.82, 1.11) | 0.55 | 0.35  (0.07, 1.90) | 0.23 | 1.01  (0.78, 1.29) | 0.96 |
| Secondary school unfinished | 1.02  (0.92, 1.13) | 0.69 | 1.04  (0.96, 1.12) | 0.38 | 0.65  (0.28, 1.52) | 0.32 | 1.02  (0.44, 2.35) | 0.96 |
| Secondary school finished | 1.00  (0.87, 1.14) | 0.96 | 1.02  (0.91, 1.15) | 0.69 | 0.64  (0.27, 1.49) | 0.30 | 0.94  (0.44, 2.03) | 0.88 |
| Secondary school above | 1.10  (0.97, 1.26) | 0.14 | 1.05  (0.95, 1.16) | 0.35 | 0.52  (0.20, 1.35) | 0.18 | 0.53  (0.17, 1.68) | 0.28 |
| **BMI** |  |  |  |  |  |  |  |  |
| <18.5 kg/m^2^ (Thin) | 1.04  (0.98, 1.09) | 0.18 | 1.05  (1.02, 1.09) | <0.01 | 1.36  (0.83, 2.23) | 0.23 | 0.79  (0.55, 1.14) | 0.21 |
| 18.5-24.9 kg/m^2^ (Normal) | **1 (reference)** |  | **1 (reference)** |  | **1 (reference)** |  | **1 (reference)** |  |
| 25.0-29.9 kg/m^2^ (Overweight) | 0.98  (0.88, 1.08) | 0.66 | 1.02  (0.94, 1.10) | 0.63 | 1.24  (0.71, 2.16) | 0.45 | 0.76  (0.54, 1.08) | 0.13 |
| >30.0 kg/m^2^ (Obese) | 1.11  (0.93, 1.31) | 0.24 | 1.15  (0.97, 1.35) | 0.11 | 1.89  (0.85, 4.18) | 0.12 | 0.44  (0.19, 1.04) | 0.06 |
| **Smoking** |  |  |  |  |  |  |  |  |
| Non-smoker | **1 (reference)** |  | **1 (reference)** |  | **1 (reference)** |  | **1 (reference)** |  |
| Current smoker | 0.84  (0.70, 1.00) | 0.05 | 1.07  (0.99, 1.15) | 0.08 | 1.12  (0.47, 2.64) | 0.80 | 0.60  (0.18, 2.02) | 0.41 |
| **Use of smokeless tobacco** |  |  |  |  |  |  |  |  |
| Does not use | **1 (reference)** |  | **1 (reference)** |  | **1 (reference)** |  | **1 (reference)** |  |
| Uses smokeless tobacco | 1.15  (0.94, 1.40) | 0.18 | 1.02  (0.93, 1.12) | 0.71 | 2.62 (0.66, 10.42) | 0.17 | 3.75 (1.04, 13.51) | 0.04 |

Abbreviations: RR, relative risk; 95% CI, 95% confidence interval; BMI, body mass index.

**Table S5.** Results of household fixed effects regression models for the impact of insurance coverage on the likelihood of reaching successive hypertension care cascade steps stratified by body mass index categories.

| **BMI: ≥25 kg/m2** | | | | | | | | |
| --- | --- | --- | --- | --- | --- | --- | --- | --- |
|  | **Screened** | | **Diagnosed** | | **Treated** | | **Controlled** | |
|  | **RR**  **(95% CI)** | **P-value** | **RR**  **(95% CI)** | **P-value** | **RR**  **(95% CI)** | **P-value** | **RR**  **(95% CI)** | **P-value** |
| **Insurance Coverage** |  |  |  |  |  |  |  |  |
| Uninsured | **1 (reference)** |  | **1 (reference)** |  | **1 (reference)** |  | **1 (reference)** |  |
| Insured | 1  (0.92, 1.09) | 0.93 | 0.94  (0.78, 1.14) | 0.54 | 1.29  (0.81, 2.03) | 0.28 | 0.72  (0.34, 1.54) | 0.40 |
| **Sex** |  |  |  |  |  |  |  |  |
| Female | **1 (reference)** |  | **1 (reference)** |  | **1 (reference)** |  | **1 (reference)** |  |
| Male | 0.97  (0.9, 1.04) | 0.42 | 0.82  (0.69, 0.96) | 0.02 | 0.89  (0.63, 1.26) | 0.52 | 0.65  (0.37, 1.14) | 0.13 |
| **Marital Status** |  |  |  |  |  |  |  |  |
| Unmarried | **1 (reference)** |  | **1 (reference)** |  | **1 (reference)** |  | **1 (reference)** |  |
| Married | 1.14  (0.99, 1.31) | 0.06 | 1.08  (0.96, 1.21) | 0.21 | 1.74  (1.01, 3) | 0.05 | 1.26  (0.68, 2.34) | 0.46 |
| **Education Level** |  |  |  |  |  |  |  |  |
| No education | **1 (reference)** |  | **1 (reference)** |  | **1 (reference)** |  | **1 (reference)** |  |
| Primary school unfinished | 1.1  (0.95, 1.28) | 0.20 | 0.95  (0.45, 2) | 0.89 | 0.45  (0.22, 0.91) | 0.03 | 0.35  (0.07, 1.76) | 0.20 |
| Primary school finished | 0.99  (0.88, 1.11) | 0.85 | 1.07  (0.91, 1.25) | 0.41 | 0.69  (0.42, 1.14) | 0.15 | 0.9 (0.18, 4.56) | 0.90 |
| Secondary school unfinished | 1.05  (0.94, 1.17) | 0.41 | 1.1  (0.92, 1.33) | 0.30 | 0.65  (0.37, 1.16) | 0.15 | 0.77  (0.23, 2.6) | 0.67 |
| Secondary school finished | 0.96  (0.83, 1.11) | 0.61 | 1.03  (0.85, 1.26) | 0.75 | 0.76  (0.39, 1.5) | 0.43 | 0.49 (0.12, 1.98) | 0.32 |
| Secondary school above | 1.09  (0.94, 1.27) | 0.24 | 1.12  (0.89, 1.41) | 0.35 | 0.68  (0.32, 1.44) | 0.32 | 0.49  (0.12, 2) | 0.32 |
| **Age Group** |  |  |  |  |  |  |  |  |
| 15-19 years | **1 (reference)** |  | **1 (reference)** |  | **1 (reference)** |  | **1 (reference)** |  |
| 20-24 years | 1.78  (1.21, 2.63) | <0.01 | 0.86  (0.66, 1.12) | 0.27 | 1.69  (0.55, 5.13) | 0.36 | 2.09  (0.65, 6.73) | 0.22 |
| 25-29 years | 2.08  (1.41, 3.07) | <0.01 | 0.83  (0.63, 1.09) | 0.19 | 1.01  (0.32, 3.22) | 0.99 | 1.41  (0.48, 4.13) | 0.53 |
| 30-34 years | 1.92  (1.29, 2.84) | <0.01 | 0.96  (0.72, 1.28) | 0.77 | 0.85  (0.25, 2.82) | 0.79 | 1.42  (0.38, 5.22) | 0.60 |
| 35-39 years | 1.99  (1.35, 2.93) | <0.01 | 0.74  (0.55, 0.98) | 0.04 | 1.13  (0.34, 3.81) | 0.84 | 0.68  (0.28, 1.64) | 0.39 |
| 40-44 years | 2.06  (1.37, 3.08) | <0.01 | 0.97  (0.74, 1.25) | 0.79 | 1.75  (0.6, 5.08) | 0.30 | 0.55  (0.21, 1.43) | 0.22 |
| 45-49 years | 2  (1.37, 2.92) | <0.01 | 1.02  (0.81, 1.3) | 0.85 | 2.49  (0.89, 6.94) | 0.08 | 1.02  (0.41, 2.54) | 0.96 |
| **Smoking** |  |  |  |  |  |  |  |  |
| Non-smoker | **1 (reference)** |  | **1 (reference)** |  | **1 (reference)** |  | **1 (reference)** |  |
| Current smoker | 0.83  (0.7, 0.97) | 0.02 | 1.09  (0.85, 1.41) | 0.49 | 1.29  (0.73, 2.3) | 0.38 | 0.71  (0.2, 2.59) | 0.61 |
| **Use of smokeless tobacco** |  |  |  |  |  |  |  |  |
| Does not use | **1 (reference)** |  | **1 (reference)** |  | **1 (reference)** |  | **1 (reference)** |  |
| Uses smokeless tobacco | 1.05  (0.87, 1.27) | 0.62 | 1.05  (0.82, 1.34) | 0.71 | 1.13  (0.61, 2.11) | 0.70 | 1.35  (0.26, 7.17) | 0.72 |
| **BMI: < 25 kg/m2** | | | | | | | | |
|  | **Screened** | | **Diagnosed** | | **Treated** | | **Controlled** | |
|  | **RR**  **(95% CI)** | **P-value** | **RR**  **(95% CI)** | **P-value** | **RR**  **(95% CI)** | **P-value** | **RR**  **(95% CI)** | **P-value** |
| **Insurance Coverage** |  |  |  |  |  |  |  |  |
| Uninsured | **1 (reference)** |  | **1 (reference)** |  | **1 (reference)** |  | **1 (reference)** |  |
| Insured | 0.97  (0.9, 1.03) | 0.32 | 0.97  (0.9, 1.04) | 0.44 | 1.39  (0.94, 2.04) | 0.10 | 0.9  (0.75, 1.08) | 0.24 |
| **Sex** |  |  |  |  |  |  |  |  |
| Female | **1 (reference)** |  | **1 (reference)** |  | **1 (reference)** |  | **1 (reference)** |  |
| Male | 0.91  (0.87, 0.96) | <0.01 | 0.93  (0.9, 0.97) | <0.01 | 0.95  (0.66, 1.36) | 0.76 | 1.12  (0.91, 1.38) | 0.29 |
| **Marital Status** |  |  |  |  |  |  |  |  |
| Unmarried | **1 (reference)** |  | **1 (reference)** |  | **1 (reference)** |  | **1 (reference)** |  |
| Married | 1.11  (1.06, 1.17) | <0.01 | 1.01  (0.97, 1.06) | 0.54 | 1.22  (0.92, 1.62) | 0.17 | 1.07  (0.81, 1.43) | 0.63 |
| **Education Level** |  |  |  |  |  |  |  |  |
| No education | **1 (reference)** |  | **1 (reference)** |  | **1 (reference)** |  | **1 (reference)** |  |
| Primary school unfinished | 1.03  (0.93, 1.14) | 0.54 | 0.99  (0.9, 1.08) | 0.78 | 0.56  (0.36, 0.87) | 0.01 | 0.7  (0.49, 1) | 0.05 |
| Primary school finished | 1.01  (0.93, 1.1) | 0.84 | 0.97  (0.9, 1.04) | 0.34 | 0.98  (0.62, 1.57) | 0.94 | 0.91  (0.55, 1.52) | 0.72 |
| Secondary school unfinished | 1.06  (1, 1.12) | 0.05 | 1.01  (0.96, 1.07) | 0.60 | 0.92  (0.65, 1.3) | 0.64 | 1.05  (0.78, 1.41) | 0.74 |
| Secondary school finished | 1.04  (0.96, 1.13) | 0.31 | 0.99  (0.93, 1.06) | 0.86 | 0.96  (0.63, 1.46) | 0.86 | 0.87  (0.54, 1.4) | 0.56 |
| Secondary school above | 1.09  (1, 1.19) | 0.04 | 1.01  (0.94, 1.08) | 0.82 | 0.88  (0.53, 1.45) | 0.62 | 1.03  (0.7, 1.51) | 0.90 |
| **Age Group** |  |  |  |  |  |  |  |  |
| 15-19 years | **1 (reference)** |  | **1 (reference)** |  | **1 (reference)** |  | **1 (reference)** |  |
| 20-24 years | 1.06  (1, 1.13) | 0.05 | 1  (0.95, 1.06) | 0.98 | 0.54  (0.38, 0.75) | <0.01 | 1.05  (0.88, 1.25) | 0.61 |
| 25-29 years | 1.1  (1.02, 1.18) | 0.01 | 0.97  (0.91, 1.03) | 0.33 | 0.75  (0.45, 1.25) | 0.27 | 1.12  (0.84, 1.51) | 0.44 |
| 30-34 years | 1.14  (1.05, 1.23) | <0.01 | 0.98  (0.92, 1.05) | 0.61 | 0.82  (0.53, 1.28) | 0.39 | 0.92  (0.71, 1.18) | 0.51 |
| 35-39 years | 1.13  (1.04, 1.24) | 0.01 | 0.99  (0.92, 1.06) | 0.77 | 0.8  (0.5, 1.3) | 0.37 | 0.83  (0.54, 1.29) | 0.41 |
| 40-44 years | 1.17  (1.08, 1.26) | <0.01 | 0.94  (0.87, 1.02) | 0.16 | 1.29  (0.84, 1.96) | 0.24 | 0.76  (0.5, 1.16) | 0.20 |
| 45-49 years | 1.14  (1.05, 1.22) | <0.01 | 0.99  (0.93, 1.04) | 0.60 | 1.3  (0.9, 1.9) | 0.17 | 0.71 (0.48, 1.05) | 0.08 |
| **Smoking** |  |  |  |  |  |  |  |  |
| Non-smoker | **1 (reference)** |  | **1 (reference)** |  | **1 (reference)** |  | **1 (reference)** |  |
| Current smoker | 0.94  (0.86, 1.03) | 0.20 | 1.03  (0.96, 1.09) | 0.45 | 0.92  (0.55, 1.55) | 0.75 | 0.51  (0.28, 0.92) | 0.02 |
| **Use of smokeless tobacco** |  |  |  |  |  |  |  |  |
| Does not use | **1 (reference)** |  | **1 (reference)** |  | **1 (reference)** |  | **1 (reference)** |  |
| Uses smokeless tobacco | 0.92  (0.84, 1.02) | 0.10 | 1  (0.92, 1.09) | 0.98 | 1.22  (0.64, 2.31) | 0.54 | 1.7  (0.91, 3.17) | 0.10 |

Abbreviations: RR, relative risk; 95% CI, 95% confidence interval; BMI, body mass index.

**Table S6**. Results of household fixed effects regression models for the impact of insurance coverage on the likelihood of reaching successive hypertension care cascade steps stratified by education levels.

| **Education level: Primary finished and below** | | | | | | | | |
| --- | --- | --- | --- | --- | --- | --- | --- | --- |
|  | **Screened** | | **Diagnosed** | | **Treated** | | **Controlled** | |
|  | **RR**  **(95% CI)** | **P-value** | **RR**  **(95% CI)** | **P-value** | **RR**  **(95% CI)** | **P-value** | **RR**  **(95% CI)** | **P-value** |
| **Insurance Coverage** |  |  |  |  |  |  |  |  |
| Uninsured | **1 (reference)** |  | **1 (reference)** |  | **1 (reference)** |  | **1 (reference)** |  |
| Insured | 0.99 (0.88, 1.11) | 0.86 | 1.02 (0.92, 1.14) | 0.66 | 1.03 (0.57, 1.85) | 0.93 | 2.66 (0.92, 7.72) | 0.07 |
| **Sex** |  |  |  |  |  |  |  |  |
| Female | **1 (reference)** |  | **1 (reference)** |  | **1 (reference)** |  | **1 (reference)** |  |
| Male | 0.86 (0.78, 0.96) | <0.01 | 0.96 (0.86, 1.08) | 0.52 | 1.26 (0.75, 2.1) | 0.38 | 1.24 (0.5, 3.1) | 0.64 |
| **Marital Status** |  |  |  |  |  |  |  |  |
| Unmarried | **1 (reference)** |  | **1 (reference)** |  | **1 (reference)** |  | **1 (reference)** |  |
| Married | 1.13 (1, 1.29) | 0.06 | 0.99 (0.89, 1.1) | 0.82 | 1.16 (0.7, 1.93) | 0.56 | 1.2 (0.63, 2.27) | 0.58 |
| **Age Group** |  |  |  |  |  |  |  |  |
| 15-19 years | **1 (reference)** |  | **1 (reference)** |  | **1 (reference)** |  | **1 (reference)** |  |
| 20-24 years | 0.89 (0.7, 1.13) | 0.33 | 1.02 (0.91, 1.15) | 0.68 | 0.48 (0.24, 0.99) | 0.05 | 1.6 (0.77, 3.32) | 0.20 |
| 25-29 years | 1.01 (0.79, 1.31) | 0.91 | 1.06 (0.88, 1.27) | 0.55 | 0.91 (0.3, 2.81) | 0.87 | 1.7 (0.73, 3.97) | 0.22 |
| 30-34 years | 1.09 (0.84, 1.4) | 0.53 | 1.1 (0.85, 1.42) | 0.48 | 0.53 (0.2, 1.39) | 0.19 | 0.64 (0.15, 2.7) | 0.54 |
| 35-39 years | 1.01 (0.8, 1.28) | 0.93 | 1.08 (0.92, 1.26) | 0.35 | 0.91 (0.42, 1.98) | 0.81 | 1.32 (0.29, 6.02) | 0.72 |
| 40-44 years | 1.07 (0.85, 1.34) | 0.56 | 0.83 (0.7, 0.99) | 0.04 | 0.8 (0.39, 1.67) | 0.56 | 0.39 (0.16, 0.95) | 0.04 |
| 45-49 years | 1.06 (0.87, 1.3) | 0.55 | 0.96 (0.84, 1.1) | 0.54 | 0.57 (0.26, 1.22) | 0.15 | 0.79 (0.42, 1.49) | 0.47 |
| **BMI** |  |  |  |  |  |  |  |  |
| <18.5 kg/m^2^ (Thin) | **1 (reference)** |  | **1 (reference)** |  | **1 (reference)** |  | **1 (reference)** |  |
| 18.5-24.9 kg/m^2^ (Normal) | 1.07 (0.97, 1.19) | 0.15 | 0.95 (0.86, 1.04) | 0.28 | 0.88 (0.57, 1.36) | 0.58 | 1.11 (0.59, 2.08) | 0.74 |
| 25.0-29.9 kg/m^2^ (Overweight) | 0.87 (0.76, 1) | 0.05 | 1.16 (0.95, 1.43) | 0.15 | 0.95 (0.57, 1.58) | 0.83 | 1.3 (0.62, 2.73) | 0.48 |
| >30.0 kg/m^2^ (Obese) | 1.18 (0.96, 1.45) | 0.11 | 1.16 (0.94, 1.43) | 0.16 | 0.86 (0.43, 1.74) | 0.68 | 0.47 (0.08, 2.76) | 0.41 |
| **Smoking** |  |  |  |  |  |  |  |  |
| Non-smoker | **1 (reference)** |  | **1 (reference)** |  | **1 (reference)** |  | **1 (reference)** |  |
| Current smoker | 0.97 (0.83, 1.14) | 0.74 | 0.96 (0.85, 1.09) | 0.53 | 0.87 (0.42, 1.8) | 0.70 | 0.28 (0.09, 0.92) | 0.04 |
| **Use of smokeless tobacco** |  |  |  |  |  |  |  |  |
| Does not use | **1 (reference)** |  | **1 (reference)** |  | **1 (reference)** |  | **1 (reference)** |  |
| Uses smokeless tobacco | 0.99 (0.87, 1.14) | 0.92 | 0.88 (0.73, 1.05) | 0.16 | 0.77 (0.4, 1.46) | 0.42 | 1.8 (0.64, 5.08) | 0.27 |
| **Education level: Secondary unfinished and above** | | | | | | | | |
|  | **Screened** | | **Diagnosed** | | **Treated** | | **Controlled** | |
|  | **RR**  **(95% CI)** | **P-value** | **RR**  **(95% CI)** | **P-value** | **RR**  **(95% CI)** | **P-value** | **RR**  **(95% CI)** | **P-value** |
| **Insurance Coverage** |  |  |  |  |  |  |  |  |
| Uninsured | **1 (reference)** |  | **1 (reference)** |  | **1 (reference)** |  | **1 (reference)** |  |
| Insured | 0.96 (0.9, 1.01) | 0.13 | 0.96 (0.89, 1.03) | 0.24 | 0.98 (0.7, 1.36) | 0.89 | 0.85 (0.6, 1.2) | 0.35 |
| **Sex** |  |  |  |  |  |  |  |  |
| Female | **1 (reference)** |  | **1 (reference)** |  | **1 (reference)** |  | **1 (reference)** |  |
| Male | 0.96 (0.92, 1) | 0.06 | 0.94 (0.89, 0.99) | 0.02 | 0.88 (0.67, 1.14) | 0.33 | 0.85 (0.61, 1.18) | 0.33 |
| **Marital Status** |  |  |  |  |  |  |  |  |
| Unmarried | **1 (reference)** |  | **1 (reference)** |  | **1 (reference)** |  | **1 (reference)** |  |
| Married | 1.13 (1.06, 1.21) | <0.01 | 1.04 (0.98, 1.1) | 0.24 | 1.55 (1.07, 2.26) | 0.02 | 0.82 (0.6, 1.13) | 0.23 |
| **Age Group** |  |  |  |  |  |  |  |  |
| 15-19 years | **1 (reference)** |  | **1 (reference)** |  | **1 (reference)** |  | **1 (reference)** |  |
| 20-24 years | 1.12 (1.03, 1.21) | 0.01 | 0.97 (0.93, 1.02) | 0.21 | 0.7 (0.44, 1.1) | 0.12 | 0.89 (0.65, 1.22) | 0.47 |
| 25-29 years | 1.18 (1.06, 1.32) | <0.01 | 0.95 (0.88, 1.02) | 0.13 | 0.59 (0.32, 1.09) | 0.09 | 0.68 (0.45, 1.04) | 0.07 |
| 30-34 years | 1.16 (1.04, 1.3) | 0.01 | 0.98 (0.91, 1.06) | 0.63 | 0.77 (0.46, 1.31) | 0.34 | 0.84 (0.54, 1.32) | 0.46 |
| 35-39 years | 1.21 (1.09, 1.34) | <0.01 | 0.89 (0.81, 0.98) | 0.02 | 0.54 (0.28, 1.04) | 0.06 | 0.71 (0.42, 1.2) | 0.20 |
| 40-44 years | 1.22 (1.09, 1.37) | <0.01 | 0.96 (0.88, 1.05) | 0.39 | 1.03 (0.62, 1.7) | 0.92 | 0.78 (0.47, 1.32) | 0.36 |
| 45-49 years | 1.23 (1.11, 1.35) | <0.01 | 1.01 (0.94, 1.08) | 0.88 | 1.18 (0.69, 2.04) | 0.54 | 0.75 (0.41, 1.38) | 0.35 |
| **BMI** |  |  |  |  |  |  |  |  |
| <18.5 kg/m^2^ (Thin) | **1 (reference)** |  | **1 (reference)** |  | **1 (reference)** |  | **1 (reference)** |  |
| 18.5-24.9 kg/m^2^ (Normal) | 1.03 (0.98, 1.09) | 0.26 | 1.09 (1.04, 1.14) | <0.01 | 1.06 (0.67, 1.67) | 0.82 | 0.82 (0.57, 1.18) | 0.29 |
| 25.0-29.9 kg/m^2^ (Overweight) | 0.99 (0.93, 1.04) | 0.61 | 1.03 (0.98, 1.08) | 0.19 | 1.05 (0.77, 1.42) | 0.77 | 0.68 (0.44, 1.07) | 0.09 |
| >30.0 kg/m^2^ (Obese) | 1.11 (1.02, 1.21) | 0.01 | 1.07 (0.97, 1.19) | 0.18 | 1.13 (0.83, 1.53) | 0.44 | 0.7 (0.4, 1.22) | 0.20 |
| **Smoking** |  |  |  |  |  |  |  |  |
| Non-smoker | **1 (reference)** |  | **1 (reference)** |  | **1 (reference)** |  | **1 (reference)** |  |
| Current smoker | 0.88 (0.81, 0.96) | 0.01 | 1.07 (0.98, 1.16) | 0.11 | 1.09 (0.63, 1.88) | 0.77 | 0.59 (0.2, 1.77) | 0.35 |
| **Use of smokeless tobacco** |  |  |  |  |  |  |  |  |
| Does not use | **1 (reference)** |  | **1 (reference)** |  | **1 (reference)** |  | **1 (reference)** |  |
| Uses smokeless tobacco | 1.01 (0.91, 1.12) | 0.89 | 0.96 (0.88, 1.05) | 0.43 | 1.27 (0.59, 2.72) | 0.55 | 1.31 (0.38, 4.53) | 0.67 |

Abbreviations: RR, relative risk; 95% CI, 95% confidence interval; BMI, body mass index.

**Table S7.** Results of household fixed effects regression models for the impact of insurance coverage on the likelihood of reaching successive hypertension care cascade steps stratified by smoking behaviors.

| **Tobacco consumption: Smoker** | | | | | | | | |
| --- | --- | --- | --- | --- | --- | --- | --- | --- |
|  | **Screened** | | **Diagnosed** | | **Treated** | | **Controlled** | |
|  | **RR**  **(95% CI)** | **P-value** | **RR**  **(95% CI)** | **P-value** | **RR**  **(95% CI)** | **P-value** | **RR**  **(95% CI)** | **P-value** |
| **Insurance Coverage** |  |  |  |  |  |  | **--^*^** | |
| Uninsured | **1 (reference)** |  | **1 (reference)** |  | **1 (reference)** |  |  |  |
| Insured | 1.16  (0.85, 1.58) | 0.36 | 0.81  (0.59, 1.12) | 0.20 | 3.95 (0.55, 28.14) | 0.17 |  |  |
| **Sex** |  |  |  |  |  |  |  |  |
| Female | **1 (reference)** |  | **1 (reference)** |  | **1 (reference)** |  |  |  |
| Male | 0.84  (0.75, 0.95) | <0.01 | 0.92  (0.8, 1.07) | 0.29 | 0.25  (0.06, 1.12) | 0.07 |  |  |
| **Marital Status** |  |  |  |  |  |  |  |  |
| Unmarried | **1 (reference)** |  | **1 (reference)** |  | **1 (reference)** |  |  |  |
| Married | 1.16  (0.97, 1.39) | 0.10 | 1.13  (0.93, 1.36) | 0.21 | 5 (0.32, 79.24) | 0.25 |  |  |
| **Age Group** |  |  |  |  |  |  |  |  |
| 15-19 years | **1 (reference)** |  | **1 (reference)** |  | **1 (reference)** |  |  |  |
| 20-24 years | 0.99  (0.79, 1.25) | 0.95 | 0.94  (0.73, 1.2) | 0.60 | 0.03  (0, 3.55) | 0.14 |  |  |
| 25-29 years | 1.25  (0.98, 1.6) | 0.07 | 0.82  (0.64, 1.04) | 0.10 | 0.42  (0, 47.2) | 0.72 |  |  |
| 30-34 years | 1.2  (0.92, 1.56) | 0.18 | 0.97  (0.71, 1.32) | 0.85 | 0.22  (0, 87.24) | 0.62 |  |  |
| 35-39 years | 1.39  (1.04, 1.87) | 0.03 | 0.73  (0.5, 1.07) | 0.11 | 0.06  (0, 5.29) | 0.22 |  |  |
| 40-44 years | 1.32  (0.97, 1.79) | 0.07 | 0.78  (0.6, 1.02) | 0.07 | 0.5 (0.01, 36.05) | 0.75 |  |  |
| 45-49 years | 1.09  (0.85, 1.41) | 0.50 | 0.89  (0.68, 1.18) | 0.42 | 3.48 (0.11, 113.23) | 0.48 |  |  |
| **Education Level** |  |  |  |  |  |  |  |  |
| No education | **1 (reference)** |  | **1 (reference)** |  | **1 (reference)** |  |  |  |
| Primary school unfinished | 1.1  (0.85, 1.42) | 0.47 | 0.84  (0.64, 1.12) | 0.24 | 0.1  (0.01, 2.04) | 0.14 |  |  |
| Primary school finished | 0.93  (0.67, 1.29) | 0.67 | 0.91  (0.66, 1.27) | 0.60 | 0.17  (0, 20.77) | 0.47 |  |  |
| Secondary school unfinished | 1.15  (0.9, 1.48) | 0.26 | 1.04  (0.83, 1.31) | 0.73 | 0.85 (0.03, 23.87) | 0.92 |  |  |
| Secondary school finished | 1.12  (0.8, 1.59) | 0.51 | 1.22  (0.9, 1.64) | 0.19 | 0.21  (0, 28.98) | 0.54 |  |  |
| Secondary school above | 1.04  (0.76, 1.43) | 0.81 | 1.38  (0.94, 2.02) | 0.10 | 0.94 (0.01, 71.86) | 0.98 |  |  |
| **BMI** |  |  |  |  |  |  |  |  |
| <18.5 kg/m^2^ (Thin) | **1 (reference)** |  | **1 (reference)** |  | **1 (reference)** |  |  |  |
| 18.5-24.9 kg/m^2^ (Normal) | 0.93  (0.8, 1.08) | 0.35 | 1.07  (0.86, 1.33) | 0.52 | 0.52  (0.08, 3.21) | 0.48 |  |  |
| 25.0-29.9 kg/m^2^ (Overweight) | 0.83  (0.69, 1.01) | 0.06 | 1.07  (0.92, 1.25) | 0.39 | 0.41  (0.07, 2.48) | 0.33 |  |  |
| >30.0 kg/m^2^ (Obese) | 1.13  (0.86, 1.47) | 0.38 | 1.19  (0.82, 1.72) | 0.37 | 3.42 (0.15, 76.81) | 0.44 |  |  |
| **Tobacco consumption: Non-smoker** | | | | | | | | |
|  | **Screened** | | **Diagnosed** | | **Treated** | | **Controlled** | |
|  | **RR**  **(95% CI)** | **P-value** | **RR**  **(95% CI)** | **P-value** | **RR**  **(95% CI)** | **P-value** | **RR**  **(95% CI)** | **P-value** |
| **Insurance Coverage** |  |  |  |  |  |  |  |  |
| Uninsured | **1 (reference)** |  | **1 (reference)** |  | **1 (reference)** |  | **1 (reference)** |  |
| Insured | 0.95  (0.9, 0.99) | 0.02 | 0.97  (0.91, 1.05) | 0.46 | 1.2  (0.92, 1.56) | 0.18 | 0.9  (0.73, 1.09) | 0.28 |
| **Sex** |  |  |  |  |  |  |  |  |
| Female | **1 (reference)** |  | **1 (reference)** |  | **1 (reference)** |  | **1 (reference)** |  |
| Male | 0.92  (0.89, 0.95) | <0.01 | 0.92  (0.87, 0.96) | <0.01 | 0.89  (0.72, 1.1) | 0.27 | 0.74  (0.56, 0.99) | 0.04 |
| **Marital Status** |  |  |  |  |  |  |  |  |
| Unmarried | **1 (reference)** |  | **1 (reference)** |  | **1 (reference)** |  | **1 (reference)** |  |
| Married | 1.09  (1.05, 1.14) | <0.01 | 1.06  (1.02, 1.1) | 0.01 | 1.22  (0.95, 1.56) | 0.12 | 1.02  (0.8, 1.31) | 0.84 |
| **Age Group** |  |  |  |  |  |  |  |  |
| 15-19 years | **1 (reference)** |  | **1 (reference)** |  | **1 (reference)** |  | **1 (reference)** |  |
| 20-24 years | 1.1  (1.03, 1.18) | 0.01 | 0.96  (0.93, 1.01) | 0.09 | 0.69  (0.52, 0.93) | 0.02 | 1.06  (0.87, 1.28) | 0.58 |
| 25-29 years | 1.17  (1.08, 1.26) | <0.01 | 0.92  (0.87, 0.99) | 0.02 | 0.68  (0.45, 1.03) | 0.07 | 0.81  (0.58, 1.12) | 0.21 |
| 30-34 years | 1.15  (1.07, 1.25) | <0.01 | 0.97  (0.89, 1.05) | 0.43 | 0.92  (0.61, 1.4) | 0.70 | 0.87  (0.65, 1.17) | 0.37 |
| 35-39 years | 1.22  (1.13, 1.32) | <0.01 | 0.9  (0.83, 0.97) | 0.01 | 0.72  (0.46, 1.14) | 0.16 | 0.81  (0.56, 1.15) | 0.23 |
| 40-44 years | 1.24  (1.13, 1.35) | <0.01 | 0.96  (0.89, 1.04) | 0.33 | 1.13  (0.78, 1.63) | 0.51 | 0.8  (0.58, 1.11) | 0.19 |
| 45-49 years | 1.21  (1.12, 1.3) | <0.01 | 1.01  (0.95, 1.07) | 0.75 | 1.25  (0.9, 1.75) | 0.19 | 0.96  (0.69, 1.33) | 0.80 |
| **Education Level** |  |  |  |  |  |  |  |  |
| No education | **1 (reference)** |  | **1 (reference)** |  | **1 (reference)** |  | **1 (reference)** |  |
| Primary school unfinished | 0.95  (0.88, 1.03) | 0.20 | 0.97  (0.81, 1.15) | 0.69 | 0.41  (0.25, 0.7) | <0.01 | 0.95  (0.63, 1.46) | 0.83 |
| Primary school finished | 1  (0.95, 1.05) | 0.96 | 1.02  (0.96, 1.08) | 0.55 | 0.99  (0.66, 1.47) | 0.95 | 1.46  (0.86, 2.49) | 0.16 |
| Secondary school unfinished | 1.03  (0.99, 1.08) | 0.14 | 1.03  (0.98, 1.09) | 0.20 | 0.58  (0.42, 0.79) | <0.01 | 1.46  (1.08, 1.95) | 0.01 |
| Secondary school finished | 1.07  (1, 1.14) | 0.04 | 1.01  (0.94, 1.07) | 0.84 | 0.64 (0.44, 0.92) | 0.02 | 1.48  (1, 2.19) | 0.05 |
| Secondary school above | 1.09  (1.02, 1.16) | 0.01 | 1.06  (0.99, 1.14) | 0.12 | 0.64  (0.43, 0.94) | 0.02 | 1.26  (0.78, 2.02) | 0.35 |
| **BMI** |  |  |  |  |  |  |  |  |
| <18.5 kg/m^2^ (Thin) | **1 (reference)** |  | **1 (reference)** |  | **1 (reference)** |  | **1 (reference)** |  |
| 18.5-24.9 kg/m^2^ (Normal) | 1.07  (1.03, 1.12) | <0.01 | 1.05  (1.02, 1.08) | <0.01 | 1.16  (0.9, 1.49) | 0.26 | 1.07  (0.87, 1.31) | 0.54 |
| 25.0-29.9 kg/m^2^ (Overweight) | 0.98  (0.94, 1.02) | 0.32 | 0.99  (0.95, 1.03) | 0.60 | 1.25  (1, 1.56) | 0.05 | 0.7  (0.51, 0.97) | 0.03 |
| >30.0 kg/m^2^ (Obese) | 1.09  (1.02, 1.16) | 0.01 | 1.02  (0.92, 1.13) | 0.70 | 1.18  (0.91, 1.52) | 0.20 | 0.69  (0.44, 1.1) | 0.12 |

Abbreviations: RR, relative risk; 95% CI, 95% confidence interval; BMI, body mass index.

**^*^ The coefficients of the household fixed effects model could not be estimated because of too few samples in that stratum who were from the same household but showed different insurance coverage and the care cascade outcome.**

**Table S8**. Results of household fixed effects regression models for the impact of insurance coverage on getting screened among the hypertensive population stratified by residence and household wealth categories.

| **Residence: Rural** | | | | | | |
| --- | --- | --- | --- | --- | --- | --- |
|  | **Lower** | | **Middle** | | **Upper** | |
|  | **RR**  **(95% CI)** | **P-value** | **RR**  **(95% CI)** | **P-value** | **RR**  **(95% CI)** | **P-value** |
| **Insurance Coverage** |  |  |  |  |  |  |
| Uninsured | **1 (reference)** |  | **1 (reference)** |  | **1 (reference)** |  |
| Insured | 1.13 (0.92, 1.39) | 0.26 | 0.94 (0.85, 1.05) | 0.27 | 0.97 (0.90, 1.05) | 0.5 |
| **Sex** |  |  |  |  |  |  |
| Female | **1 (reference)** |  | **1 (reference)** |  | **1 (reference)** |  |
| Male | 0.88 (0.79, 0.98) | 0.02 | 0.89 (0.83, 0.95) | <0.01 | 0.92 (0.87, 0.98) | 0.01 |
| **Age Group** |  |  |  |  |  |  |
| 15-19 years | **1 (reference)** |  | **1 (reference)** |  | **1 (reference)** |  |
| 20-24 years | 1.05 (0.89, 1.25) | 0.55 | 0.99 (0.88, 1.11) | 0.88 | 1.02 (0.93, 1.12) | 0.66 |
| 25-29 years | 1.16 (0.96, 1.40) | 0.13 | 1.04 (0.91, 1.19) | 0.53 | 1.09 (0.98, 1.22) | 0.12 |
| 30-34 years | 1.17 (0.97, 1.41) | 0.11 | 1.13 (0.99, 1.29) | 0.08 | 1.14 (1.03, 1.27) | 0.01 |
| 35-39 years | 1.11 (0.93, 1.34) | 0.24 | 1.04 (0.92, 1.18) | 0.51 | 1.19 (1.06, 1.34) | <0.01 |
| 40-44 years | 1.16 (0.96, 1.39) | 0.12 | 1.03 (0.90, 1.17) | 0.71 | 1.22 (1.08, 1.39) | <0.01 |
| 45-49 years | 1.13 (0.93, 1.37) | 0.22 | 1.03 (0.88, 1.19) | 0.74 | 1.23 (1.09, 1.37) | <0.01 |
| **Marital Status** |  |  |  |  |  |  |
| Unmarried | **1 (reference)** |  | **1 (reference)** |  | **1 (reference)** |  |
| Married | 1.38 (1.19, 1.59) | <0.01 | 1.20 (1.1, 1.31) | <0.01 | 1.08 (1.00, 1.17) | 0.05 |
| **Education Level** |  |  |  |  |  |  |
| No education | **1 (reference)** |  | **1 (reference)** |  | **1 (reference)** |  |
| Primary school unfinished | 0.95 (0.80, 1.13) | 0.58 | 0.94 (0.83, 1.06) | 0.31 | 1.00 (0.85, 1.17) | 1.00 |
| Primary school finished | 0.93 (0.77, 1.12) | 0.46 | 0.95 (0.84, 1.07) | 0.38 | 1.03 (0.96, 1.12) | 0.41 |
| Secondary school unfinished | 1.10 (0.97, 1.26) | 0.15 | 1.02 (0.93, 1.12) | 0.64 | 1.10 (1.02, 1.19) | 0.01 |
| Secondary school finished | 1.29 (1.03, 1.62) | 0.03 | 0.93 (0.79, 1.10) | 0.40 | 1.10 (1.00, 1.22) | 0.04 |
| Secondary school above | 1.07 (0.80, 1.43) | 0.66 | 1.10 (0.92, 1.32) | 0.28 | 1.09 (0.98, 1.20) | 0.10 |
| **BMI** |  |  |  |  |  |  |
| <18.5 kg/m^2^ (Thin) | 1.08 (0.96, 1.21) | 0.20 | 1.02 (0.93, 1.11) | 0.72 | 1.06 (1.00, 1.14) | 0.07 |
| 18.5-24.9 kg/m^2^ (Normal) | **1 (reference)** |  | **1 (reference)** |  | **1 (reference)** |  |
| 25.0-29.9 kg/m^2^ (Overweight) | 1.00 (0.86, 1.16) | 0.97 | 0.98 (0.92, 1.05) | 0.66 | 1.01 (0.95, 1.08) | 0.80 |
| >30.0 kg/m^2^ (Obese) | 1.14 (0.86, 1.49) | 0.36 | 1.03 (0.92, 1.15) | 0.65 | 1.09 (1.00, 1.18) | 0.05 |
| **Smoking** |  |  |  |  |  |  |
| Non-smoker | **1 (reference)** |  | **1 (reference)** |  | **1 (reference)** |  |
| Current smoker | 1.20 (0.93, 1.55) | 0.16 | 1.00 (0.85, 1.19) | 0.98 | 0.85 (0.75, 0.96) | 0.01 |
| **Use of smokeless tobacco** |  |  |  |  |  |  |
| Does not use | **1 (reference)** |  | **1 (reference)** |  | **1 (reference)** |  |
| Uses smokeless tobacco | 0.75 (0.58, 0.98) | 0.03 | 0.92 (0.76, 1.12) | 0.42 | 1.10 (0.97, 1.24) | 0.14 |
| **Residence: Urban** | | | | | | |
|  | **Lower** | | **Middle** | | **Upper** | |
|  | **RR**  **(95% CI)** | **P-value** | **RR**  **(95% CI)** | **P-value** | **RR**  **(95% CI)** | **P-value** |
| **Insurance Coverage** |  |  |  |  |  |  |
| Uninsured | **1 (reference)** |  | **1 (reference)** |  | **1 (reference)** |  |
| Insured | 0.89 (0.77, 1.03) | 0.11 | 0.99 (0.91, 1.08) | 0.87 | 0.98 (0.90, 1.08) | 0.72 |
| **Sex** |  |  |  |  |  |  |
| Female | **1 (reference)** |  | **1 (reference)** |  | **1 (reference)** |  |
| Male | 0.83 (0.75, 0.93) | <0.01 | 0.92 (0.85, 1.01) | 0.07 | 1.03 (0.94, 1.12) | 0.54 |
| **Age Group** |  |  |  |  |  |  |
| 15-19 years | **1 (reference)** |  | **1 (reference)** |  | **1 (reference)** |  |
| 20-24 years | 1.14 (0.91, 1.42) | 0.25 | 1.35 (1.09, 1.67) | 0.01 | 0.95 (0.80, 1.13) | 0.55 |
| 25-29 years | 1.35 (0.95, 1.92) | 0.1 | 1.34 (1.06, 1.69) | 0.01 | 1.13 (0.94, 1.35) | 0.20 |
| 30-34 years | 1.24 (0.92, 1.66) | 0.16 | 1.37 (1.08, 1.73) | 0.01 | 0.93 (0.72, 1.21) | 0.60 |
| 35-39 years | 1.26 (0.99, 1.62) | 0.07 | 1.45 (1.15, 1.84) | <0.01 | 1.02 (0.81, 1.29) | 0.84 |
| 40-44 years | 1.24 (0.98, 1.56) | 0.07 | 1.61 (1.21, 2.15) | <0.01 | 0.98 (0.79, 1.22) | 0.84 |
| 45-49 years | 1.19 (0.97, 1.47) | 0.09 | 1.41 (1.11, 1.79) | 0.01 | 1.05 (0.87, 1.27) | 0.63 |
| **Marital Status** |  |  |  |  |  |  |
| Unmarried | **1 (reference)** |  | **1 (reference)** |  | **1 (reference)** |  |
| Married | 1.18 (1.01, 1.37) | 0.03 | 1.09 (0.99, 1.2) | 0.09 | 1.22 (1.05, 1.43) | 0.01 |
| **Education Level** |  |  |  |  |  |  |
| No education | **1 (reference)** |  | **1 (reference)** |  | **1 (reference)** |  |
| Primary school unfinished | 0.97 (0.79, 1.18) | 0.74 | 1.20 (0.95, 1.52) | 0.13 | 0.94 (0.60, 1.47) | 0.78 |
| Primary school finished | 0.92 (0.80, 1.07) | 0.27 | 1.10 (0.94, 1.29) | 0.22 | 0.93 (0.78, 1.11) | 0.43 |
| Secondary school unfinished | 1.07 (0.92, 1.23) | 0.39 | 1.08 (0.94, 1.24) | 0.26 | 0.96 (0.83, 1.12) | 0.63 |
| Secondary school finished | 1.13 (0.91, 1.41) | 0.28 | 1.09 (0.90, 1.32) | 0.36 | 0.96 (0.82, 1.13) | 0.65 |
| Secondary school above | 1.34 (1.01, 1.78) | 0.04 | 1.07 (0.91, 1.25) | 0.41 | 1.08 (0.92, 1.27) | 0.36 |
| **BMI** |  |  |  |  |  |  |
| <18.5 kg/m^2^ (Thin) | 1.20 (1.01, 1.41) | 0.04 | 1.06 (0.91, 1.23) | 0.45 | 1.05 (0.94, 1.17) | 0.35 |
| 18.5-24.9 kg/m^2^ (Normal) | **1 (reference)** |  | **1 (reference)** |  | **1 (reference)** |  |
| 25.0-29.9 kg/m^2^ (Overweight) | 1.06 (0.91, 1.24) | 0.43 | 0.86 (0.75, 0.98) | 0.02 | 0.97 (0.88, 1.07) | 0.57 |
| >30.0 kg/m^2^ (Obese) | 1.25 (1.03, 1.51) | 0.02 | 1.04 (0.91, 1.19) | 0.55 | 1.13 (0.98, 1.29) | 0.09 |
| **Smoking** |  |  |  |  |  |  |
| Non-smoker | **1 (reference)** |  | **1 (reference)** |  | **1 (reference)** |  |
| Current smoker | 1.01 (0.86, 1.17) | 0.94 | 0.83 (0.69, 1.01) | 0.06 | 0.82 (0.71, 0.96) | 0.01 |
| **Use of smokeless tobacco** |  |  |  |  |  |  |
| Does not use | **1 (reference)** |  | **1 (reference)** |  | **1 (reference)** |  |
| Uses smokeless tobacco | 1.03 (0.89, 1.19) | 0.70 | 0.94 (0.75, 1.19) | 0.63 | 1.15 (0.92, 1.44) | 0.21 |

Abbreviations: RR, relative risk; 95% CI, 95% confidence interval; BMI, body mass index.

**Table S9**. Results of household fixed effects regression models for the impact of insurance coverage on being diagnosed among the hypertensive population who have received screening stratified by residence and household wealth categories.

| **Residence: Rural** | | | | | | |
| --- | --- | --- | --- | --- | --- | --- |
|  | **Lower** | | **Middle** | | **Upper** | |
|  | **RR**  **(95% CI)** | **P-value** | **RR**  **(95% CI)** | **P-value** | **RR**  **(95% CI)** | **P-value** |
| **Insurance Coverage** |  |  |  |  |  |  |
| Uninsured | **1 (reference)** |  | **1 (reference)** |  | **1 (reference)** |  |
| Insured | 1.04 (0.97, 1.11) | 0.31 | 0.96 (0.81, 1.13) | 0.59 | 1.00 (0.92, 1.08) | 0.91 |
| **Sex** |  |  |  |  |  |  |
| Female | **1 (reference)** |  | **1 (reference)** |  | **1 (reference)** |  |
| Male | 0.98 (0.91, 1.05) | 0.50 | 0.90 (0.83, 0.97) | 0.01 | 0.89 (0.84, 0.94) | <0.01 |
| **Age Group** |  |  |  |  |  |  |
| 15-19 years | **1 (reference)** |  | **1 (reference)** |  | **1 (reference)** |  |
| 20-24 years | 1.07 (0.97, 1.18) | 0.19 | 1.04 (0.94, 1.16) | 0.44 | 1.00 (0.93, 1.08) | 0.93 |
| 25-29 years | 1.06 (0.90, 1.24) | 0.49 | 0.96 (0.84, 1.11) | 0.60 | 0.98 (0.89, 1.08) | 0.73 |
| 30-34 years | 0.96 (0.84, 1.10) | 0.55 | 1.00 (0.87, 1.14) | 0.95 | 1.04 (0.92, 1.17) | 0.55 |
| 35-39 years | 1.10 (0.94, 1.30) | 0.24 | 1.03 (0.92, 1.16) | 0.62 | 1.04 (0.91, 1.17) | 0.59 |
| 40-44 years | 1.00 (0.88, 1.14) | 0.99 | 0.96 (0.85, 1.08) | 0.49 | 0.99 (0.88, 1.11) | 0.84 |
| 45-49 years | 1.04 (0.90, 1.21) | 0.60 | 1.00 (0.92, 1.10) | 0.92 | 1.05 (0.94, 1.17) | 0.42 |
| **Marital Status** |  |  |  |  |  |  |
| Unmarried | **1 (reference)** |  | **1 (reference)** |  | **1 (reference)** |  |
| Married | 1.03 (0.94, 1.12) | 0.54 | 0.96 (0.87, 1.05) | 0.37 | 1.03 (0.93, 1.13) | 0.6 |
| **Education Level** |  |  |  |  |  |  |
| No education | **1 (reference)** |  | **1 (reference)** |  | **1 (reference)** |  |
| Primary school unfinished | 0.95 (0.78, 1.17) | 0.65 | 1.02 (0.86, 1.22) | 0.80 | 1.00 (0.88, 1.14) | 0.97 |
| Primary school finished | 0.85 (0.71, 1.02) | 0.09 | 1.02 (0.90, 1.14) | 0.80 | 0.98 (0.90, 1.06) | 0.60 |
| Secondary school unfinished | 1.05 (0.95, 1.16) | 0.38 | 1.04 (0.93, 1.16) | 0.49 | 0.98 (0.92, 1.06) | 0.64 |
| Secondary school finished | 1.01 (0.93, 1.10) | 0.83 | 1.07 (0.94, 1.21) | 0.33 | 0.95 (0.84, 1.08) | 0.46 |
| Secondary school above | 0.92 (0.74, 1.14) | 0.47 | 1.12 (0.94, 1.34) | 0.20 | 0.98 (0.89, 1.08) | 0.75 |
| **BMI** |  |  |  |  |  |  |
| <18.5 kg/m^2^ (Thin) | 1.03 (0.95, 1.13) | 0.46 | 0.96 (0.87, 1.06) | 0.47 | 1.07 (1.01, 1.13) | 0.02 |
| 18.5-24.9 kg/m^2^ (Normal) | **1 (reference)** |  | **1 (reference)** |  | **1 (reference)** |  |
| 25.0-29.9 kg/m^2^ (Overweight) | 0.97 (0.87, 1.07) | 0.53 | 1.00 (0.93, 1.07) | 0.96 | 1.07 (1.01, 1.13) | 0.03 |
| >30.0 kg/m^2^ (Obese) | 0.94 (0.73, 1.23) | 0.67 | 1.07 (0.97, 1.17) | 0.19 | 1.02 (0.94, 1.10) | 0.70 |
| **Smoking** |  |  |  |  |  |  |
| Non-smoker | **1 (reference)** |  | **1 (reference)** |  | **1 (reference)** |  |
| Current smoker | 0.87 (0.75, 1.01) | 0.06 | 1.10 (0.97, 1.25) | 0.14 | 1.05 (0.93, 1.19) | 0.41 |
| **Use of smokeless tobacco** |  |  |  |  |  |  |
| Does not use | **1 (reference)** |  | **1 (reference)** |  | **1 (reference)** |  |
| Uses smokeless tobacco | 1.13 (0.95, 1.35) | 0.17 | 0.93 (0.79, 1.10) | 0.41 | 0.98 (0.86, 1.13) | 0.83 |
| **Residence: Urban** | | | | | | |
|  | **Lower** | | **Middle** | | **Upper** | |
|  | **RR**  **(95% CI)** | **P-value** | **RR**  **(95% CI)** | **P-value** | **RR**  **(95% CI)** | **P-value** |
| **Insurance Coverage** |  |  |  |  |  |  |
| Uninsured | **1 (reference)** |  | **1 (reference)** |  | **1 (reference)** |  |
| Insured | 1.00 (0.89, 1.13) | 0.95 | 1.06 (0.95, 1.19) | 0.31 | 0.88 (0.76, 1.02) | 0.09 |
| **Sex** |  |  |  |  |  |  |
| Female | **1 (reference)** |  | **1 (reference)** |  | **1 (reference)** |  |
| Male | 1.06 (0.90, 1.25) | 0.49 | 0.87 (0.79, 0.95) | <0.01 | 0.94 (0.84, 1.05) | 0.28 |
| **Age Group** |  |  |  |  |  |  |
| 15-19 years | **1 (reference)** |  | **1 (reference)** |  | **1 (reference)** |  |
| 20-24 years | 0.99 (0.87, 1.13) | 0.90 | 0.97 (0.88, 1.08) | 0.61 | 0.86 (0.75, 0.98) | 0.02 |
| 25-29 years | 1.04 (0.90, 1.19) | 0.61 | 1.02 (0.87, 1.18) | 0.84 | 0.75 (0.63, 0.89) | <0.01 |
| 30-34 years | 1.37 (0.98, 1.90) | 0.07 | 1.01 (0.85, 1.20) | 0.93 | 0.84 (0.71, 0.99) | 0.04 |
| 35-39 years | 1.00 (0.84, 1.20) | 0.96 | 0.80 (0.64, 1.01) | 0.06 | 0.72 (0.60, 0.86) | <0.01 |
| 40-44 years | 0.89 (0.68, 1.16) | 0.38 | 0.98 (0.86, 1.11) | 0.72 | 0.86 (0.72, 1.03) | 0.10 |
| 45-49 years | 1.03 (0.89, 1.20) | 0.65 | 1.01 (0.89, 1.14) | 0.92 | 0.86 (0.76, 0.97) | 0.01 |
| **Marital Status** |  |  |  |  |  |  |
| Unmarried | **1 (reference)** |  | **1 (reference)** |  | **1 (reference)** |  |
| Married | 0.97 (0.87, 1.07) | 0.54 | 1.04 (0.95, 1.13) | 0.38 | 1.14 (1.03, 1.25) | 0.01 |
| **Education Level** |  |  |  |  |  |  |
| No education | **1 (reference)** |  | **1 (reference)** |  | **1 (reference)** |  |
| Primary school unfinished | 1.12 (0.90, 1.4) | 0.30 | 0.69 (0.38, 1.27) | 0.23 | 1.30 (0.74, 2.29) | 0.37 |
| Primary school finished | 1.02 (0.86, 1.21) | 0.79 | 1.03 (0.92, 1.15) | 0.64 | 1.04 (0.88, 1.24) | 0.64 |
| Secondary school unfinished | 1.02 (0.86, 1.21) | 0.82 | 1.03 (0.92, 1.15) | 0.60 | 1.00 (0.90, 1.11) | 0.99 |
| Secondary school finished | 0.96 (0.76, 1.21) | 0.75 | 0.99 (0.87, 1.14) | 0.93 | 0.96 (0.86, 1.08) | 0.52 |
| Secondary school above | 0.89 (0.69, 1.16) | 0.39 | 1.06 (0.92, 1.21) | 0.44 | 1.06 (0.94, 1.20) | 0.36 |
| **BMI** |  |  |  |  |  |  |
| <18.5 kg/m^2^ (Thin) | 1.22 (1.03, 1.45) | 0.02 | 1.04 (0.95, 1.13) | 0.40 | 1.07 (0.97, 1.17) | 0.18 |
| 18.5-24.9 kg/m^2^ (Normal) | **1 (reference)** |  | **1 (reference)** |  | **1 (reference)** |  |
| 25.0-29.9 kg/m^2^ (Overweight) | 1.04 (0.85, 1.26) | 0.72 | 0.94 (0.86, 1.03) | 0.18 | 1.01 (0.94, 1.10) | 0.75 |
| >30.0 kg/m^2^ (Obese) | 1.08 (0.89, 1.31) | 0.44 | 0.95 (0.73, 1.23) | 0.70 | 1.20 (1.05, 1.38) | 0.01 |
| **Smoking** |  |  |  |  |  |  |
| Non-smoker | **1 (reference)** |  | **1 (reference)** |  | **1 (reference)** |  |
| Current smoker | 0.83 (0.66, 1.04) | 0.10 | 1.12 (0.97, 1.29) | 0.14 | 1.08 (0.95, 1.22) | 0.24 |
| **Use of smokeless tobacco** |  |  |  |  |  |  |
| Does not use | **1 (reference)** |  | **1 (reference)** |  | **1 (reference)** |  |
| Uses smokeless tobacco | 0.92 (0.70, 1.21) | 0.54 | 0.98 (0.81, 1.18) | 0.84 | 0.96 (0.81, 1.13) | 0.61 |

Abbreviations: RR, relative risk; 95% CI, 95% confidence interval; BMI, body mass index.

**Table S10**. Results of household fixed effects regression models for the impact of insurance coverage on getting treated among the hypertensive population who have been diagnosed stratified by residence and household wealth categories.

| **Residence: Rural** | | | | | | |
| --- | --- | --- | --- | --- | --- | --- |
|  | **Lower** | | **Middle** | | **Upper** | |
|  | **RR**  **(95% CI)** | **P-value** | **RR**  **(95% CI)** | **P-value** | **RR**  **(95% CI)** | **P-value** |
| **Insurance Coverage** |  |  |  |  |  |  |
| Uninsured | **1 (reference)** |  | **1 (reference)** |  | **1 (reference)** |  |
| Insured | 3.13 (0.8, 12.21) | 0.10 | 1.18 (0.63, 2.20) | 0.61 | 0.78 (0.53, 1.13) | 0.18 |
| **Sex** |  |  |  |  |  |  |
| Female | **1 (reference)** |  | **1 (reference)** |  | **1 (reference)** |  |
| Male | 1.14 (0.52, 2.49) | 0.74 | 1.62 (0.87, 3.00) | 0.13 | 0.82 (0.57, 1.18) | 0.29 |
| **Age Group** |  |  |  |  |  |  |
| 15-19 years | **1 (reference)** |  | **1 (reference)** |  | **1 (reference)** |  |
| 20-24 years | 0.40 (0.17, 0.95) | 0.04 | 0.50 (0.24, 1.03) | 0.06 | 0.79 (0.53, 1.17) | 0.24 |
| 25-29 years | 1.00 (0.27, 3.65) | 1.00 | 0.82 (0.32, 2.12) | 0.68 | 0.68 (0.44, 1.06) | 0.09 |
| 30-34 years | 0.77 (0.29, 2.02) | 0.60 | 1.67 (0.63, 4.46) | 0.30 | 0.65 (0.38, 1.11) | 0.12 |
| 35-39 years | 0.33 (0.10, 1.11) | 0.07 | 1.45 (0.65, 3.21) | 0.36 | 0.65 (0.38, 1.11) | 0.11 |
| 40-44 years | 0.49 (0.18, 1.32) | 0.16 | 1.53 (0.64, 3.66) | 0.34 | 0.89 (0.56, 1.40) | 0.61 |
| 45-49 years | 0.47 (0.19, 1.16) | 0.10 | 1.23 (0.55, 2.73) | 0.61 | 1.24 (0.80, 1.91) | 0.34 |
| **Marital Status** |  |  |  |  |  |  |
| Unmarried | **1 (reference)** |  | **1 (reference)** |  | **1 (reference)** |  |
| Married | 1.36 (0.67, 2.78) | 0.40 | 1.23 (0.69, 2.20) | 0.48 | 1.47 (1.05, 2.06) | 0.02 |
| **Education Level** |  |  |  |  |  |  |
| No education | **1 (reference)** |  | **1 (reference)** |  | **1 (reference)** |  |
| Primary school unfinished | 0.62 (0.25, 1.56) | 0.31 | 0.60 (0.29, 1.22) | 0.16 | 0.25 (0.13, 0.48) | <0.01 |
| Primary school finished | 0.95 (0.37, 2.47) | 0.92 | 0.83 (0.43, 1.63) | 0.60 | 0.78 (0.50, 1.22) | 0.28 |
| Secondary school unfinished | 0.38 (0.19, 0.73) | 0.00 | 0.87 (0.51, 1.47) | 0.59 | 0.56 (0.38, 0.82) | <0.01 |
| Secondary school finished | 0.52 (0.21, 1.30) | 0.16 | 0.91 (0.41, 2.00) | 0.81 | 0.66 (0.42, 1.03) | 0.07 |
| Secondary school above | 1.53 (0.39, 5.93) | 0.54 | 0.35 (0.13, 0.99) | 0.05 | 0.57 (0.34, 0.97) | 0.04 |
| **BMI** |  |  |  |  |  |  |
| <18.5 kg/m^2^ (Thin) | 0.78 (0.45, 1.35) | 0.37 | 1.37 (0.95, 1.99) | 0.09 | 0.96 (0.62, 1.47) | 0.84 |
| 18.5-24.9 kg/m^2^ (Normal) | **1 (reference)** |  | **1 (reference)** |  | **1 (reference)** |  |
| 25.0-29.9 kg/m^2^ (Overweight) | 0.82 (0.18, 3.62) | 0.79 | 0.95 (0.59, 1.53) | 0.84 | 0.99 (0.73, 1.34) | 0.97 |
| >30.0 kg/m^2^ (Obese) | 0.48 (0.11, 2.11) | 0.33 | 0.65 (0.21, 2.00) | 0.45 | 0.81 (0.56, 1.18) | 0.27 |
| **Smoking** |  |  |  |  |  |  |
| Non-smoker | **1 (reference)** |  | **1 (reference)** |  | **1 (reference)** |  |
| Current smoker | 0.65 (0.15, 2.77) | 0.56 | 0.41 (0.18, 0.94) | 0.04 | 1.51 (0.85, 2.69) | 0.16 |
| **Use of smokeless tobacco** |  |  |  |  |  |  |
| Does not use | **1 (reference)** |  | **1 (reference)** |  | **1 (reference)** |  |
| Uses smokeless tobacco | 1.51 (0.29, 7.93) | 0.63 | 1.41 (0.62, 3.22) | 0.41 | 1.36 (0.68, 2.73) | 0.38 |
| **Residence: Urban** | | | | | | |
|  | **Lower** | | **Middle** | | **Upper** | |
|  | **RR**  **(95% CI)** | **P-value** | **RR**  **(95% CI)** | **P-value** | **RR**  **(95% CI)** | **P-value** |
| **Insurance Coverage** |  |  |  |  |  |  |
| Uninsured | **1 (reference)** |  | **1 (reference)** |  | **1 (reference)** |  |
| Insured | 1.06 (0.62, 1.83) | 0.83 | 2.24 (1.02, 4.94) | 0.05 | 1.15 (0.66, 1.99) | 0.62 |
| **Sex** |  |  |  |  |  |  |
| Female | **1 (reference)** |  | **1 (reference)** |  | **1 (reference)** |  |
| Male | 0.54 (0.25, 1.15) | 0.11 | 0.77 (0.41, 1.43) | 0.41 | 1.02 (0.76, 1.38) | 0.88 |
| **Age Group** |  |  |  |  |  |  |
| 15-19 years | **1 (reference)** |  | **1 (reference)** |  | **1 (reference)** |  |
| 20-24 years | 0.35 (0.11, 1.10) | 0.07 | 0.69 (0.27, 1.74) | 0.43 | 1.57 (0.62, 3.96) | 0.34 |
| 25-29 years | 0.28 (0.07, 1.10) | 0.07 | 1.47 (0.45, 4.78) | 0.52 | 1.14 (0.31, 4.25) | 0.85 |
| 30-34 years | 2.33 (0.56, 9.79) | 0.25 | 1.92 (0.5, 7.44) | 0.34 | 1.05 (0.27, 4.06) | 0.94 |
| 35-39 years | 1.12 (0.23, 5.42) | 0.88 | 2.68 (0.78, 9.28) | 0.12 | 0.53 (0.11, 2.68) | 0.45 |
| 40-44 years | 0.94 (0.23, 3.78) | 0.93 | 4.91 (1.49, 16.15) | 0.01 | 2.13 (0.63, 7.19) | 0.22 |
| 45-49 years | 1.50 (0.36, 6.19) | 0.57 | 3.02 (0.94, 9.73) | 0.06 | 2.32 (0.67, 8.10) | 0.19 |
| **Marital Status** |  |  |  |  |  |  |
| Unmarried | **1 (reference)** |  | **1 (reference)** |  | **1 (reference)** |  |
| Married | 0.57 (0.21, 1.56) | 0.27 | 0.63 (0.32, 1.24) | 0.18 | 1.90 (0.79, 4.60) | 0.15 |
| **Education Level** |  |  |  |  |  |  |
| No education | **1 (reference)** |  | **1 (reference)** |  | **1 (reference)** |  |
| Primary school unfinished | 0.25 (0.06, 0.99) | 0.05 | 2.55 (0.84, 7.71) | 0.10 | 0.28 (0.04, 1.93) | 0.19 |
| Primary school finished | 1.25 (0.11, 14.8) | 0.86 | 0.62 (0.26, 1.50) | 0.29 | 1.34 (0.51, 3.54) | 0.55 |
| Secondary school unfinished | 0.99 (0.27, 3.59) | 0.99 | 0.99 (0.50, 1.96) | 0.97 | 0.58 (0.16, 2.11) | 0.41 |
| Secondary school finished | 0.67 (0.13, 3.39) | 0.63 | 0.88 (0.38, 2.04) | 0.76 | 0.41 (0.11, 1.48) | 0.17 |
| Secondary school above | 0.75 (0.17, 3.30) | 0.70 | 1.67 (0.58, 4.78) | 0.34 | 0.33 (0.09, 1.16) | 0.08 |
| **BMI** |  |  |  |  |  |  |
| <18.5 kg/m^2^ (Thin) | 0.90 (0.30, 2.71) | 0.86 | 1.55 (0.07, 3.43) | 0.28 | 2.44 (0.92, 6.45) | 0.07 |
| 18.5-24.9 kg/m^2^ (Normal) | **1 (reference)** |  | **1 (reference)** |  | **1 (reference)** |  |
| 25.0-29.9 kg/m^2^ (Overweight) | 2.10 (1.13, 3.91) | 0.02 | 2.46 (1.37, 4.40) | <0.01 | 1.02 (0.69, 1.51) | 0.94 |
| >30.0 kg/m^2^ (Obese) | 3.17 (0.93, 10.86) | 0.07 | 2.29 (1.26, 4.16) | 0.01 | 1.34 (0.82, 2.17) | 0.24 |
| **Smoking** |  |  |  |  |  |  |
| Non-smoker | **1 (reference)** |  | **1 (reference)** |  | **1 (reference)** |  |
| Current smoker | 1.47 (0.62, 3.51) | 0.39 | 1.56 (0.70, 3.52) | 0.28 | 1.54 (0.55, 4.34) | 0.41 |
| **Use of smokeless tobacco** |  |  |  |  |  |  |
| Does not use | **1 (reference)** |  | **1 (reference)** |  | **1 (reference)** |  |
| Uses smokeless tobacco | 1.70 (0.46, 6.23) | 0.42 | 0.80 (0.21, 3.05) | 0.74 | 0.34 (0.11, 1.02) | 0.05 |

Abbreviations: RR, relative risk; 95% CI, 95% confidence interval; BMI, body mass index.

**Table S11**. Results of household fixed effects regression models for the impact of insurance coverage on having hypertension controlled among the hypertensive population who have been treated stratified by residence and household wealth categories.

| **Residence: Rural** | | | | | | |
| --- | --- | --- | --- | --- | --- | --- |
|  | **Lower** | | **Middle** | | **Upper** | |
|  | **RR**  **(95% CI)** | **P-value** | **RR**  **(95% CI)** | **P-value** | **RR**  **(95% CI)** | **P-value** |
| **Insurance Coverage** |  |  |  |  |  |  |
| Uninsured | **1 (reference)** |  | **1 (reference)** |  | **1 (reference)** |  |
| Insured | 1.10 (0.58, 2.08) | 0.77 | 0.94 (0.65, 1.36) | 0.75 | 0.74 (0.53, 1.05) | 0.09 |
| **Sex** |  |  |  |  |  |  |
| Female | **1 (reference)** |  | **1 (reference)** |  | **1 (reference)** |  |
| Male | 0.87 (0.48, 1.58) | 0.66 | 2.19 (1.07, 4.46) | 0.03 | 0.73 (0.50, 1.06) | 0.09 |
| **Age Group** |  |  |  |  |  |  |
| 15-19 years | **1 (reference)** |  | **1 (reference)** |  | **1 (reference)** |  |
| 20-24 years | 0.66 (0.33, 1.33) | 0.25 | 0.56 (0.35, 0.91) | 0.02 | 1.44 (1.02, 2.04) | 0.04 |
| 25-29 years | 0.47 (0.20, 1.10) | 0.08 | 0.53 (0.26, 1.09) | 0.09 | 0.77 (0.44, 1.35) | 0.37 |
| 30-34 years | 0.56 (0.24, 1.33) | 0.19 | 0.25 (0.10, 0.63) | <0.01 | 1.29 (0.78, 2.14) | 0.32 |
| 35-39 years | 0.98 (0.45, 2.13) | 0.96 | 0.39 (0.15, 0.99) | 0.05 | 1.28 (0.59, 2.76) | 0.53 |
| 40-44 years | 0.39 (0.19, 0.83) | 0.01 | 0.23 (0.08, 0.61) | <0.01 | 0.79 (0.41, 1.54) | 0.49 |
| 45-49 years | 0.61 (0.30, 1.26) | 0.18 | 0.18 (0.06, 0.51) | <0.01 | 1.03 (0.62, 1.71) | 0.91 |
| **Marital Status** |  |  |  |  |  |  |
| Unmarried | **1 (reference)** |  | **1 (reference)** |  | **1 (reference)** |  |
| Married | 1.94 (0.95, 3.98) | 0.07 | 1.43 (0.87, 2.35) | 0.16 | 0.90 (0.62, 1.31) | 0.58 |
| **Education Level** |  |  |  |  |  |  |
| No education | **1 (reference)** |  | **1 (reference)** |  | **1 (reference)** |  |
| Primary school unfinished | 0.65 (0.28, 1.52) | 0.32 | 0.91 (0.48, 1.73) | 0.77 | 0.39 (0.13, 1.15) | 0.09 |
| Primary school finished | 0.79 (0.37, 1.70) | 0.55 | 0.78 (0.24, 2.54) | 0.68 | 0.78 (0.37, 1.64) | 0.51 |
| Secondary school unfinished | 1.20 (0.61, 2.35) | 0.60 | 0.50 (0.28, 0.91) | 0.02 | 1.12 (0.65, 1.95) | 0.69 |
| Secondary school finished | 0.93 (0.19, 4.50) | 0.92 | 0.52 (0.25, 1.10) | 0.09 | 0.92 (0.45, 1.86) | 0.82 |
| Secondary school above | 1.00 (0.38, 2.66) | 1.00 | 0.30 (0.12, 0.76) | 0.01 | 0.72 (0.31, 1.69) | 0.45 |
| **BMI** |  |  |  |  |  |  |
| <18.5 kg/m^2^ (Thin) | 1.22 (0.73, 2.02) | 0.45 | 0.98 (0.66, 1.44) | 0.91 | 0.85 (0.50, 1.42) | 0.53 |
| 18.5-24.9 kg/m^2^ (Normal) | **1 (reference)** |  | **1 (reference)** |  | **1 (reference)** |  |
| 25.0-29.9 kg/m^2^ (Overweight) | 0.16 (0.02, 1.42) | 0.10 | 0.68 (0.44, 1.04) | 0.08 | 0.81 (0.55, 1.20) | 0.30 |
| >30.0 kg/m^2^ (Obese) | 0.95 (0.15, 6.19) | 0.96 | 0.65 (0.27, 1.60) | 0.35 | 0.57 (0.27, 1.21) | 0.15 |
| **Smoking** |  |  |  |  |  |  |
| Non-smoker | **1 (reference)** |  | **1 (reference)** |  | **1 (reference)** |  |
| Current smoker | 0.30 (0.10, 0.85) | 0.02 | 0.09 (0.02, 0.40) | <0.01 | 0.33 (0.11, 1.00) | 0.05 |
| **Use of smokeless tobacco** |  |  |  |  |  |  |
| Does not use | **1 (reference)** |  | **1 (reference)** |  | **1 (reference)** |  |
| Uses smokeless tobacco | 2.81 (0.81, 9.73) | 0.10 | 7.04 (1.74, 28.37) | 0.01 | 3.12 (0.91, 10.64) | 0.07 |
| **Residence: Urban** | | | | | | |
|  | **Lower** | | **Middle** | | **Upper** | |
|  | **RR**  **(95% CI)** | **P-value** | **RR**  **(95% CI)** | **P-value** | **RR**  **(95% CI)** | **P-value** |
| **Insurance Coverage** | --^*^ | |  |  |  |  |
| Uninsured |  |  | **1 (reference)** |  | **1 (reference)** |  |
| Insured |  |  | 2.39 (0.89, 6.42) | 0.08 | 0.72 (0.31, 1.68) | 0.45 |
| **Sex** |  |  |  |  |  |  |
| Female |  |  | **1 (reference)** |  | **1 (reference)** |  |
| Male |  |  | 0.58 (0.18, 1.89) | 0.36 | 0.63 (0.32, 1.25) | 0.19 |
| **Age Group** |  |  |  |  |  |  |
| 15-19 years |  |  | **1 (reference)** |  | **1 (reference)** |  |
| 20-24 years |  |  | 1.18 (0.72, 1.95) | 0.51 | 0.69 (0.13, 3.81) | 0.67 |
| 25-29 years |  |  | 0.77 (0.23, 2.53) | 0.66 | 0.44 (0.08, 2.34) | 0.34 |
| 30-34 years |  |  | 0.73 (0.20, 2.76) | 0.65 | 0.30 (0.03, 2.72) | 0.29 |
| 35-39 years |  |  | 0.70 (0.17, 2.87) | 0.62 | 0.32 (0.04, 2.31) | 0.26 |
| 40-44 years |  |  | 1.69 (0.38, 7.59) | 0.49 | 0.37 (0.05, 3.03) | 0.36 |
| 45-49 years |  |  | 1.04 (0.11, 9.46) | 0.97 | 0.45 (0.07, 2.96) | 0.40 |
| **Marital Status** |  |  |  |  |  |  |
| Unmarried |  |  | **1 (reference)** |  | **1 (reference)** |  |
| Married |  |  | 0.74 (0.30, 1.81) | 0.50 | 0.79 (0.22, 2.81) | 0.72 |
| **Education Level** |  |  |  |  |  |  |
| No education |  |  | **1 (reference)** |  | **1 (reference)** |  |
| Primary school unfinished |  |  | 1.60 (0.47, 5.50) | 0.45 | 1.76 (0.08, 40.92) | 0.73 |
| Primary school finished |  |  | 0.82 (0.05, 13.48) | 0.89 | 6.05 (0.95, 38.7) | 0.06 |
| Secondary school unfinished |  |  | 3.94 (1.19, 13.05) | 0.03 | 1.13 (0.18, 7.01) | 0.90 |
| Secondary school finished |  |  | 8.56 (1.11, 65.80) | 0.04 | 0.97 (0.10, 9.29) | 0.98 |
| Secondary school above |  |  | 7.38 (1.28, 42.39) | 0.03 | 0.72 (0.11, 4.86) | 0.74 |
| **BMI** |  |  |  |  |  |  |
| <18.5 kg/m^2^ (Thin) |  |  | 1.08 (0.61, 1.90) | 0.79 | 0.54 (0.09, 3.12) | 0.49 |
| 18.5-24.9 kg/m^2^ (Normal) |  |  | **1 (reference)** |  | **1 (reference)** |  |
| 25.0-29.9 kg/m^2^ (Overweight) |  |  | 0.80 (0.29, 2.24) | 0.67 | 0.66 (0.23, 1.86) | 0.43 |
| >30.0 kg/m^2^ (Obese) |  |  | 1.07 (0.26, 4.36) | 0.93 | 0.77 (0.28, 2.10) | 0.61 |
| **Smoking** |  |  |  |  |  |  |
| Non-smoker |  |  | **1 (reference)** |  | **1 (reference)** |  |
| Current smoker |  |  | 0.15 (0.02, 1.42) | 0.10 | 3.14 (0.53, 18.46) | 0.21 |
| **Use of smokeless tobacco** |  |  |  |  |  |  |
| Does not use |  |  | **1 (reference)** |  | **1 (reference)** |  |
| Uses smokeless tobacco |  |  | 0.80 (0.03, 22.98) | 0.90 | 0.14 (0.02, 1.02) | 0.05 |

Abbreviations: RR, relative risk; 95% CI, 95% confidence interval; BMI, body mass index.

**^*^ The coefficients of the household fixed effects model could not be estimated because of too few samples in that stratum who were from the same household but showed different insurance coverage and the care cascade outcome.**

**Table S12.** Results of district-level fixed effects regression models for the impact of insurance coverage on the likelihood of reaching successive hypertension care cascade steps.

|  | **Screened** | | **Diagnosed** | | **Treated** | | **Controlled** | |
| --- | --- | --- | --- | --- | --- | --- | --- | --- |
|  | **RR**  **(95% CI)** | **P-value** | **RR**  **(95% CI)** | **P-value** | **RR**  **(95% CI)** | **P-value** | **RR**  **(95% CI)** | **P-value** |
| **Insurance Coverage** |  |  |  |  |  |  |  |  |
| Uninsured | **1 (reference)** |  | **1 (reference)** |  | **1 (reference)** |  | **1 (reference)** |  |
| Insured | 1.02  (1.00, 1.04) | 0.02 | 1.00  (0.97, 1.04) | 0.96 | 1.02  (0.94, 1.09) | 0.68 | 1.00  (0.93, 1.08) | 0.94 |
| **Sex** |  |  |  |  |  |  |  |  |
| Female | **1 (reference)** |  | **1 (reference)** |  | **1 (reference)** |  | **1 (reference)** |  |
| Male | 0.81  (0.80, 0.83) | <0.01 | 0.77  (0.75, 0.80) | <0.01 | 0.97  (0.90, 1.04) | 0.37 | 0.86  (0.79, 0.93) | <0.01 |
| **Age Group** |  |  |  |  |  |  |  |  |
| 15-19 years | **1 (reference)** |  | **1 (reference)** |  | **1 (reference)** |  | **1 (reference)** |  |
| 20-24 years | 1.14  (1.09, 1.20) | <0.01 | 0.90  (0.86, 0.94) | <0.01 | 0.74  (0.60, 0.90) | <0.01 | 0.99  (0.89, 1.10) | 0.86 |
| 25-29 years | 1.20  (1.14, 1.26) | <0.01 | 0.82  (0.78, 0.87) | <0.01 | 0.81  (0.67, 0.98) | 0.03 | 0.91  (0.83, 1.01) | 0.08 |
| 30-34 years | 1.18  (1.12, 1.24) | <0.01 | 0.80  (0.76, 0.85) | <0.01 | 0.96  (0.80, 1.15) | 0.67 | 0.81  (0.73, 0.91) | <0.01 |
| 35-39 years | 1.20  (1.14, 1.26) | <0.01 | 0.77  (0.72, 0.81) | <0.01 | 1.16  (0.97, 1.39) | 0.11 | 0.70  (0.63, 0.79) | <0.01 |
| 40-44 years | 1.22  (1.16, 1.28) | <0.01 | 0.80  (0.75, 0.84) | <0.01 | 1.59  (1.33, 1.90) | <0.01 | 0.63  (0.56, 0.71) | <0.01 |
| 45-49 years | 1.24  (1.18, 1.30) | <0.01 | 0.83  (0.78, 0.87) | <0.01 | 1.75  (1.47, 2.09) | <0.01 | 0.63  (0.56, 0.70) | <0.01 |
| **Residence** |  |  |  |  |  |  |  |  |
| Urban | **1 (reference)** |  | **1 (reference)** |  | **1 (reference)** |  | **1 (reference)** |  |
| Rural | 0.92  (0.91, 0.94) | <0.01 | 0.99  (0.96, 1.02) | 0.49 | 0.98  (0.91, 1.05) | 0.53 | 0.96  (0.91, 1.02) | 0.24 |
| **Household Wealth** |  |  |  |  |  |  |  |  |
| Lower | **1 (reference)** |  | **1 (reference)** |  | **1 (reference)** |  | **1 (reference)** |  |
| Middle | 1.07  (1.05, 1.10) | <0.01 | 1.03  (1, 1.07) | 0.04 | 1.06  (0.98, 1.14) | 0.13 | 0.98  (0.92, 1.05) | 0.57 |
| Upper | 1.13  (1.11, 1.16) | <0.01 | 1.04  (1, 1.07) | 0.07 | 1.15  (1.06, 1.26) | <0.01 | 1.02  (0.94, 1.10) | 0.64 |
| **Marital Status** |  |  |  |  |  |  |  |  |
| Unmarried | **1 (reference)** |  | **1 (reference)** |  | **1 (reference)** |  | **1 (reference)** |  |
| Married | 1.11  (1.07, 1.14) | <0.01 | 1.02  (0.99, 1.06) | 0.22 | 1.04  (0.96, 1.13) | 0.34 | 0.96  (0.89, 1.02) | 0.20 |
| **Education Level** |  |  |  |  |  |  |  |  |
| No education | **1 (reference)** |  | **1 (reference)** |  | **1 (reference)** |  | **1 (reference)** |  |
| Primary school unfinished | 0.99  (0.95, 1.03) | 0.68 | 1.02  (0.98, 1.08) | 0.33 | 0.97  (0.89, 1.06) | 0.51 | 0.98  (0.89, 1.08) | 0.70 |
| Primary school finished | 1.03  (1.01, 1.06) | 0.02 | 1.02  (0.98, 1.06) | 0.34 | 1.01  (0.91, 1.11) | 0.92 | 0.98  (0.89, 1.09) | 0.73 |
| Secondary school unfinished | 1.07  (1.05, 1.09) | <0.01 | 1.07  (1.03, 1.10) | <0.01 | 0.92  (0.86, 0.98) | 0.01 | 0.98  (0.92, 1.05) | 0.54 |
| Secondary school finished | 1.09  (1.06, 1.13) | <0.01 | 1.01  (0.97, 1.06) | 0.58 | 0.96  (0.86, 1.07) | 0.45 | 0.96  (0.84, 1.09) | 0.50 |
| Secondary school above | 1.15  (1.12, 1.18) | <0.01 | 1.08  (1.03, 1.13) | <0.01 | 0.95  (0.86, 1.06) | 0.36 | 1.07  (0.96, 1.20) | 0.23 |
| **BMI** |  |  |  |  |  |  |  |  |
| <18.5 kg/m^2^ (Thin) | 0.99  (0.97, 1.02) | 0.48 | 1.10  (1.07, 1.14) | <0.01 | 0.99  (0.9, 1.08) | 0.77 | 1.12  (1.06, 1.19) | <0.01 |
| 18.5-24.9 kg/m^2^ (Normal) | **1 (reference)** |  | **1 (reference)** |  | **1 (reference)** |  | **1 (reference)** |  |
| 25.0-29.9 kg/m^2^ (Overweight) | 1.01  (0.99, 1.02) | 0.54 | 0.98  (0.95, 1.01) | 0.16 | 1.32  (1.23, 1.40) | <0.01 | 0.83  (0.77, 0.89) | <0.01 |
| >30.0 kg/m^2^ (Obese) | 1.05  (1.03, 1.08) | <0.01 | 1.01  (0.96, 1.05) | 0.83 | 1.67  (1.56, 1.79) | <0.01 | 0.81  (0.75, 0.89) | <0.01 |
| **Smoking** |  |  |  |  |  |  |  |  |
| Non-smoker | **1 (reference)** |  | **1 (reference)** |  | **1 (reference)** |  | **1 (reference)** |  |
| Current smoker | 1.01  (0.97, 1.04) | 0.75 | 1.08  (1.02, 1.14) | 0.01 | 0.93  (0.8, 1.07) | 0.31 | 0.9  (0.75, 1.08) | 0.25 |
| **Use of smokeless tobacco** |  |  |  |  |  |  |  |  |
| Does not use | **1 (reference)** |  | **1 (reference)** |  | **1 (reference)** |  | **1 (reference)** |  |
| Uses smokeless tobacco | 0.95  (0.92, 0.99) | 0.02 | 0.88  (0.83, 0.93) | <0.01 | 1.02  (0.88, 1.19) | 0.80 | 1.00  (0.84, 1.21) | 0.96 |

Abbreviations: RR, relative risk; 95% CI, 95% confidence interval; BMI, body mass index.
